# Histone decrotonylation plays a distinct role in HIV latency

**DOI:** 10.1126/sciadv.aec0149

**Published:** 2026-04-10

**Authors:** Xiaoyi Li, Dajiang Li, Yuyang Tang, Marie Nearing, Benjamin Varco-Merth, Hongjie Chen, Davey Smith, Sara Gianella, Nancie M. Archin, Afam A. Okoye, Satya Dandekar, David M. Margolis, Venkat R. Chirasani, Ryan H. Gumpper, Guochun Jiang

**Affiliations:** ^1^ UNC HIV Cure Center, University of North Carolina, Chapel Hill, NC 27599, USA.; ^2^ Institute of Global Health & Infectious Diseases, University of North Carolina, Chapel Hill, NC 27599, USA.; ^3^ Department of Medical Microbiology and Immunology, University of California, Davis, CA 95616, USA.; ^4^ Vaccine & Gene Therapy Institute and Oregon National Primate Research Center, Oregon Health and Science University, Beaverton, OR 97006, USA.; ^5^ Department of Medicine, University of California, San Diego, CA 92093, USA.; ^6^ Department of Microbiology and Immunology, University of North Carolina School of Medicine, University of North Carolina at Chapel Hill, Chapel Hill, NC 27516, USA.; ^7^ Department of Biochemistry and Biophysics, University of North Carolina, Chapel Hill, NC 27599, USA.; ^8^ R. L. Juliano Structural Bioinformatics Core, University of North Carolina, Chapel Hill, NC 27599, USA.; ^9^ UNC Eshelman School of Pharmacy, University of North Carolina, Chapel Hill, NC 27599, USA.

## Abstract

The role of epigenetic regulation in HIV latency remains incompletely understood. We show that histone deacetylase 3 (HDAC3) inhibits trans-activator of transcription (Tat)-mediated HIV transcription through histone decrotonylation (HDCR), independent of deacetylase activity. Chemical biology approaches identified selective HDCR inhibitors (HDCRis) that reverse HIV latency with minimal impact on other histone acylations. Although HDAC2, HDAC3, and HDAC8 exhibit HDCR activity, genetic and chemical studies reveal that the HDCRi citarinostat is selective for HDAC3 and HDAC8. Molecular docking suggests that HDCRi binds outside the zinc-binding pocket, distinct from the classical HDAC inhibitor vorinostat (SAHA, suberoylanilide hydroxamic acid). Key residues (arginine-265, arginine-301, glutamine-113, and aspartic acid–57) are essential for HDCR selectivity, as their mutation abolishes HDCR activity and increases histone crotonylation without altering other acylation marks. Citarinostat increases histone crotonylation at the HIV long terminal repeat, robustly activating HIV transcription in cell lines, primary CD4^+^ T cells, and brain microglia from simian immunodeficiency virus–infected nonhuman primates and participants enrolled in the Last Gift rapid research autopsy cohort, highlighting HDCR as a promising therapeutic target for HIV latency.

## Teaser

Histone decrotonylation regulates HIV latency in T cells and brain microglia.

## INTRODUCTION

Despite the remarkable success of antiretroviral therapy (ART) in suppressing active viral replication and transforming HIV-1 (HIV) into a manageable chronic condition, the stable latently infected, replication-competent HIV reservoir remains a major barrier to HIV eradication (*1*–*3*). These reservoirs evade the host immune system and are resistant to antiviral therapy. To address this challenge, latency reversal agents (LRAs) have been explored to reactivate viral gene expression in ART-suppressed people with HIV (PWH). This reactivation aims to render infected cells susceptible to viral cytopathic effects or immune-mediated clearance, potentially facilitating a cure for HIV (*4*, *5*). Although LRAs have been shown to stimulate viral gene expression in vitro and in vivo successfully, they typically only reactivate a small subset of latent proviruses and have not led to substantial reductions in reservoir size. As a result, the translation of this strategy into clinical practice remains elusive (*6*–*8*).

Epigenetic regulation plays a critical role in maintaining HIV latency (*9*), with mechanisms such as histone modifications, DNA methylation, and chromatin remodeling tightly controlling the transcriptional silencing of integrated HIV proviruses (*10*). Among these mechanisms, histone deacetylases (HDACs) and histone methyltransferases (HMTs), rather than DNA methylation, appear to be critical regulators of HIV latency in primary CD4^+^ T cells (*11*). Inhibiting HDAC or HMT has shown potential for reactivating latent HIV, and several inhibitors targeting these enzymes have been explored as potential LRAs (*12*–*14*). Although it is well established that HDAC inhibitors (HDACis) can disrupt latent HIV in vivo (*14*, *15*), clinical trials have largely failed to achieve robust latency reversal. As with other LRAs, HDACi alone cannot reduce viral reservoirs (*14*, *16*), underscoring the complexity of epigenetic regulation of HIV latency.

Recent studies have revealed that the histone acetyltransferase (HAT) p300 also catalyzes histone crotonylation (*17*–*19*), a modification that has been shown to stimulate gene transcription (*17*, *20*). In parallel, HDACs also function as histone decrotonylase (*21*, *22*), contributing to the regulation of histone crotonylation. While histone acetylation is widely studied and recognized for its role in transcription regulation, histone crotonylation has emerged as a potent mark for transcriptional activation, and HDACs play a critical role in removing crotonyl marks (*23*). Recent findings suggest that p300-catalyzed histone crotonylation stimulates gene transcription more effectively than acetylation alone (*17*, *24*–*26*), raising the possibility that targeting the crotonylation pathway could improve latency reversal efforts. However, the role of histone decrotonylation (HDCR) in HIV transcription and latency in human immune cells remains unclear, largely because of the overlapping acylation activities of the HDCR enzymes.

In this study, we investigate the role of HDCR in HIV latency and transcription. We demonstrate the unique role of HDCR in regulating HIV latency in T cells and possibly non–T cells, paving the way for understanding the mechanisms of HDCR in the establishment of HIV latency and developing selective and potent HDCR inhibitor (HDCRi) as a next generation of epigenetic LRAs.

## RESULTS

### Crotonylation is associated with HIV transcription and latency reversal

**Fig. 1.**
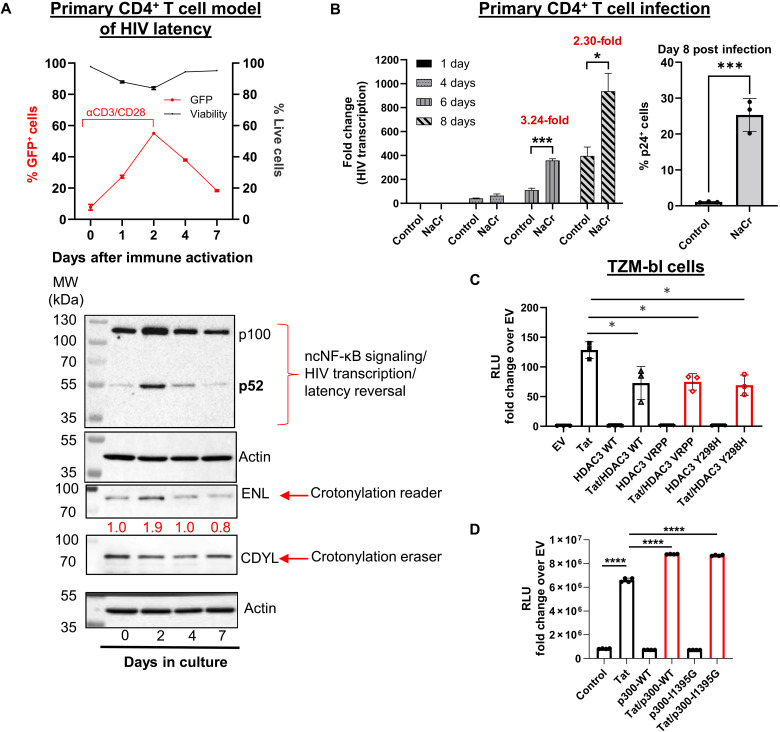
Crotonylation is associated with HIV transcription and latency reversal. (**A**) Primary CD4^+^ T cell model of latency was activated with anti-CD3/CD28 Dynabeads. The percentage of GFP^+^ cells was measured by flow cytometry (top). The levels of the ncNF-κB components p100 and p52, as well as ENL and CDYL were measured by Western blot (bottom) (*n* = 3). The relative intensity was shown below with results normalized to dimethyl sulfoxide (DMSO) control. MW, molecular weight. (**B**) Primary CD4^+^ T cells from HIV-negative donors were infected with HIV-1 (SF162) and treated with or without 40 mM NaCr (*n* = 3). HIV transcription levels were measured by reverse transcription quantitative polymerase chain reaction (RT-qPCR) targeting the HIV LTR, with results normalized to day 1 (left). The percentage of p24^+^ cells was measured by HIV-Flow assay (right). (**C**) TZM-bl luciferase reporter cells were transfected with empty vector (EV), Tat, wild-type (WT) HDAC3, or mutant HDAC3 (HDAC3-VRPP or HDAC3-Y298H), or Tat in combination with WT or mutant HDAC3 for 2 days (*n* = 3). (**D**) A similar luciferase assay was performed with WT p300 and mutant p300-I1395G. Tat-induced HIV transcription was measured using a luciferase assay. Results are expressed as relative light units (RLU) and normalized to the EV control (*n* = 4). The *P* values were determined using two-way analysis of variance (ANOVA) with multiple comparisons [(B), left], two-tailed unpaired Student’s *t* test [(B), right], or one-way ANOVA with multiple comparisons (C). Error bars represent SD; *P < 0.05; ***P < 0.001; ****P < 0.0001.

Building on our prior work (*23*, *27*, *28*), we investigated crotonylation signaling in HIV transcription using a primary CD4^+^ T cell model of latency, where green fluorescent protein (GFP) serves as a transcriptional reporter for HIV (*29*). Following treatment with anti-CD3/CD28 Dynabeads, GFP^+^ cells at day 2 gradually declined as HIV reentered latency. As cleavage of p100 into p52 activates noncanonical nuclear factor κB (ncNF-κB) signaling, important in driving HIV transcription (*23*, *28*), we monitored ncNF-κB activation as a surrogate for HIV transcription. Consistent with GFP dynamics, ncNF-κB activation (via p100 cleavage) also peaked at day 2 and subsequently declined. The temporal expression of ENL, a crotonylation reader and YEATS domain protein (*25*), mirrored HIV transcription kinetics. Conversely, levels of the crotonylation erasing enzyme chromodomain Y‐like (CDYL) showed a trend of decreasing within 2 to 4 days postactivation but rebounding by day 7, coinciding with HIV latency reestablishment (Fig. 1A). These findings highlight the association between crotonylation signaling and HIV transcription regulation, aligning with our earlier studies (*23*).

Our previous work demonstrated that the supplementation of NaCr transiently elevates cellular crotonyl–coenzyme A (CoA) levels, inducing histone crotonylation and reactivating latent HIV in T cells (*23*, *28*). Extending this to HIV-infected primary T cells, we observed a two- to threefold increase in HIV long terminal repeat (LTR)–driven transcription after 6 to 8 days of NaCr treatment compared to untreated controls (Fig. 1B, left). Moreover, HIV p24 flow cytometry demonstrated a significant increase in the frequency of p24^+^ cells in the NaCr-treated group compared to untreated controls at day 8 (Fig. 1B, right). Previous studies have reported that HDAC3 mutants, such as VRPP and Tyr^298^→His (Y298H), have decrotonylation activity but lack deacetylation activity (*21*, *30*). We found that, similar to wild-type (WT) HDAC3, these HDAC activity–defective HDAC3 mutants were sufficient to suppress trans-activator of transcription (Tat)-driven HIV transcription (Fig. 1C). However, we found that both WT and mutated HDAC1 had minimal activity in the inhibition of Tat transactivation of HIV (fig. S1A). On the other hand, we generated a p300 I1395G mutant, which has been shown to retain histone crotonyltransferase (HCT) activity but has impaired HAT activity (*31*). The I1395G mutant enhanced Tat-driven transactivation to a similar extent as WT p300 (Fig. 1D), indicating that HCT stimulates Tat transactivation of HIV without HAT activity.

Together, these results suggest that histone crotonylation promotes HIV transcription and that HDCR activity contributes to HIV latency. However, because the HDAC3 mutants retain residual (de)acylation activity toward other modifications (e.g., butyrylation, propionylation, and l-lactylation) (*20*), the specific role of HDCR in HIV transcription and latency requires further definition.

### Many HDACis also act as histone decrotonylase inhibitors

**Fig. 2.**
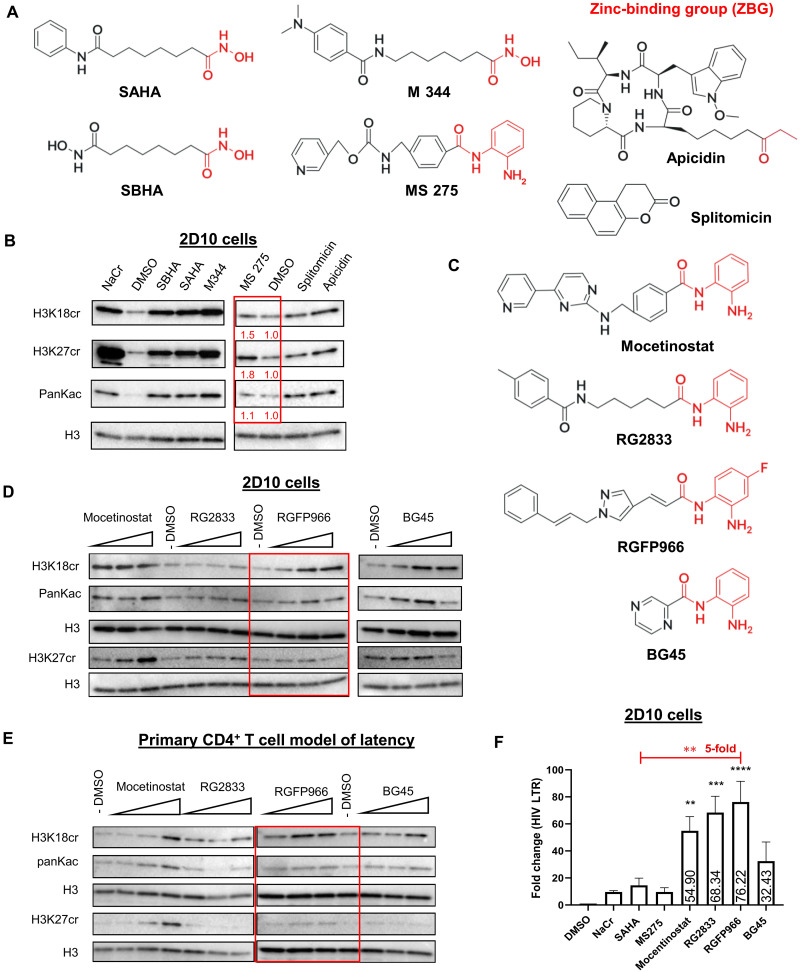
HDACis also inhibit HDCR. (**A**) Structures of some HDACis with or without ZBG. (**B**) Representative Western blotting of indicated histone marks in 2D10 cells treated with 40 mM NaCr, 2.5 μm SBHA, 350 nM SAHA, 5 μm M344, 500 nM MS275, 100 μm splitomicin, and 100 nM apicidin for 1 hour. The relative intensity was shown below with results normalized to DMSO control. PanKac; pan lysine acetylation. (**C**) Structures of some HDACis with unique benzamide ZBG. (**D**) Representative Western blotting of indicated histone marks in 2D10 cells treated with mocetinostat (100 nM, 1 μM, and 10 μM), RG2833 (100 nM, 1 μM, and 10 μM), RGFP966 (1, 5, and 10 μM), and BG45 (5, 15, and 25 μM) for 2 hours. (**E**) Representative Western blotting of indicated histone marks in primary CD4^+^ T cell model of latency treated with same doses of mocetinostat, RG2833, RGFP966, and BG45 for 4 hours. (**F**) 2D10 cells were treated with indicated HDACis for 24 hours. HIV transcription levels were measured by RT-qPCR targeting the HIV-1 LTR, with results normalized to DMSO control (*n* = 5). For (B), (D), and (E), DMSO was used as a negative control. Results are representative of three biological replicate experiments with consistent results. The *P* values were determined using one-way ANOVA with multiple comparisons (F). Error bars represent SD; **P < 0.01; ***P < 0.001; ****P < 0.0001.

Emerging evidence has shown that some HDACis could have HDCRi activity in cancer cell studies (*32*). We hypothesized that certain currently available HDACi may exhibit selective activity toward HDCR over HDAC. A typical HDACi consists of three components: a zinc-binding group (ZBG), a cap region, and a linker that connects these two parts (*33*). Although both the cap and linker are involved in the activities of HDACis, the ZBG plays a unique role in targeting the active site of HDAC activity in the enzyme, thus determining the potency and selectivity of the HDACis (*34*). Therefore, for initial screening, we selected several HDACis with diverse ZBG, including vorinostat [suberoylanilide hydroxamic acid (SAHA)] (*14*, *23*), suberoyl bishydroxamic acid (SBHA), M344, MS275, and apicidin (*14*, *35*–*37*), some of which have known activity in HIV latency reversal (*14*, *37*, *38*) (Fig. 2A). The crotonylation inducer NaCr was also included. In comparison, we included splitomicin (*39*), an HDACi that lacks a ZBG (Fig. 2A). We found that most of these compounds, including NaCr and SAHA, induced both histone crotonylation and acetylation in 2D10 cells, even within a few hours of stimulation. Therefore, we called them dual-targeting inhibitors thereafter. However, MS275 demonstrated notable selectivity by enhancing both histone H3 lysine 18 (H3K18) and histone H3 lysine 27 (H3K27) crotonylation while causing minimal alterations in acetylation (Fig. 2B).

We noticed that MS275 is HDAC3-selective and contains a unique benzamide ZBG. On the basis of these findings, we expanded our analysis to other HDAC3 inhibitors (HDAC3is) that contain unique benzamide ZBG (Fig. 2C). Our results indicated that overall, HDAC3is with benzamide ZBGs, such as RG2833 (*40*), RGFP966 (*41*), and BG45 (*42*), preferentially enhanced histone crotonylation over acetylation in both 2D10 cells (Fig. 2D) and a primary CD4^+^ T cell model of latency (Fig. 2E). However, a high concentration of mocetinostat (*37*) induced high levels of acetylation. Notably, among these HDAC3is, RGFP966, a well-characterized specific HDAC3i, demonstrated a dose-dependent increase in crotonylation with minimal effects on acetylation. These results suggest that RGFP966 may selectively inhibit histone decrotonylase activity. To assess the impact of these HDACis on HIV transcription, we measured HIV transcription in 2D10 cells by reverse transcription quantitative polymerase chain reaction (RT-qPCR) at 4, 6, and 24 hours posttreatment. While minimal changes in transcription were observed in RGFP966-treated cells at 4 hours, a moderate increase in latency reversal was observed at 6 hours (fig. S1B). At 24 hours posttreatment, RGFP966 induced a significant latency reversal, achieving a fivefold increase compared to SAHA (Fig. 2F). Given that SAHA inhibits both deacetylation and decrotonylation and RGFP966 appears to act as a selective HDCRi, our findings suggest that benzamide ZBG containing HDCR3 inhibitor may have unique activities to selectively target HDCR over HDAC, yielding greater efficacy and the potential for fewer off-target or toxicity effects while reversing HIV latency.

### Fluorine substitution on the benzamide ZBG of HDAC3i affects HDCRi activities in reversing HIV latency

**Fig. 3.**
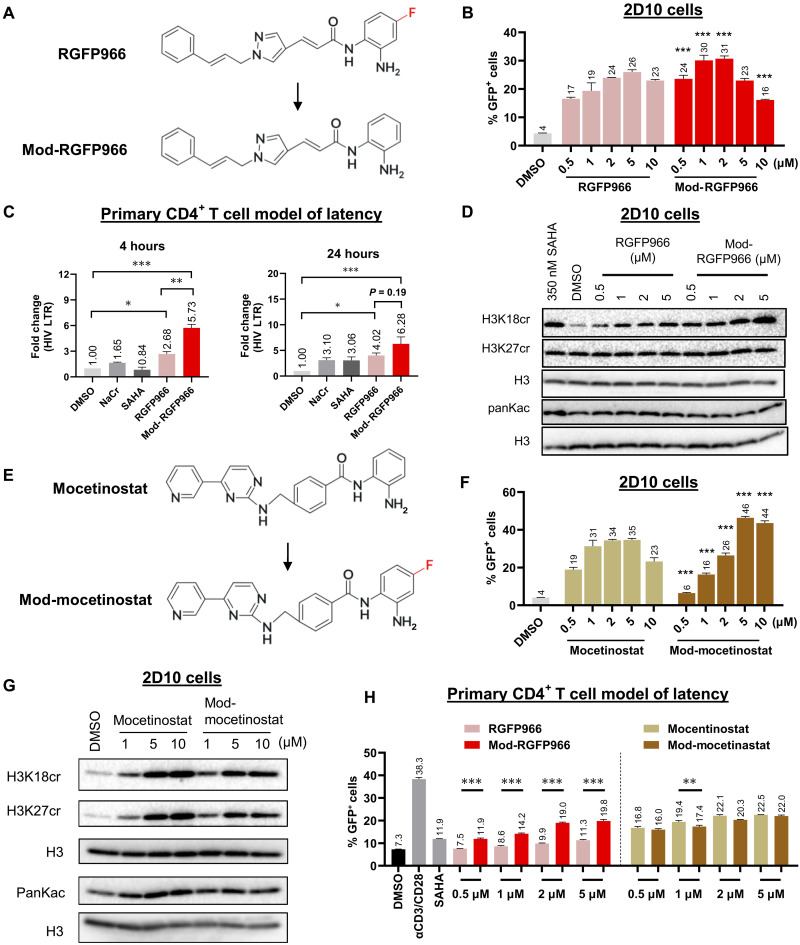
Fluorine substitution on the benzamide ZBG of HDAC3i affects HDCR activities in HIV latency models. (**A**) Chemical structures of RGFP966 and mod-RGFP966. (**B**) 2D10 cells were treated with RGFP966 and mod-RGFP966 at the indicated concentrations for 24 hours. The expression of GFP was measured by flow cytometry (*n* = 3). Statistical analysis demonstrated significant differences between RGFP966 and mod-RGFP966. (**C**) Primary CD4^+^ T cell model of latency was treated with 40 mM NaCr, 350 nM SAHA, 10 μM RGFP966, and 10 μM mod-RGFP966 for 4 or 24 hours. HIV transcription levels were measured by RT-qPCR targeting the HIV-1 LTR, with results normalized to DMSO control (*n* = 5). (**D**) 2D10 cells were treated with RGFP966 and mod-RGFP966 for 4 hours. DMSO was used as a negative control. Histones were extracted for total histone H3 and posttranslational modification (PTM) analysis using Western blot. (**E**) Chemical structures of mocetinostat and mod-mocetinostat. (**F**) 2D10 cells were treated with mocetinostat and mod-mocetinostat at the indicated concentrations for 24 hours. The expression of GFP was measured by flow cytometry (*n* = 3). Statistical analysis demonstrated significant differences between mocetinostat and mod-mocetinostat. (**G**) 2D10 cells were treated with mocetinostat and mod-mocetinostat for 4 hours. DMSO was used as a negative control. Histones were extracted for total histone H3 and PTM analysis using Western blot. (**H**) Primary CD4^+^ T cell model of latency was treated with the indicated HDACis for 24 hours, and the expression of GFP was measured by flow cytometry (*n* = 3). For (D) and (G), DMSO was used as a negative control. Results are representative of three biological replicate experiments with consistent results. The *P* values were determined using one-way ANOVA with multiple comparisons [(B), (C), (F), and (H)]. Error bars represent SD; *P < 0.05; **P < 0.01; ***P < 0.001.

Our initial hypothesis suggested that the benzamide ZBG of HDAC3i determines the selectivity for HDCR over HDAC activity. To further investigate the structural basis of this selectivity, we studied a unique fluorine modification on the benzamide ZBG ring of RGFP966. Given fluorine’s established role in modulating molecular stability, binding affinity, and reactivity (*43*, *44*), we hypothesized that fluorine may play some role in the HDCR selectivity and latency-reversing activity. We speculated that removing fluorine might diminish the HDCR selectivity and activity of RGFP966. To test this, we removed fluorine from its benzamide ZBG and synthesized a modified RGFP966 (mod-RGFP966) (Fig. 3A). Contrary to our hypothesis, the mod-RGFP966 exhibited enhanced potency in reversing HIV latency at concentrations below 2 μM (Fig. 3B), alongside reduced cytotoxicity compared to the parent compound (fig. S2A). At higher concentrations (>5 μM), both compounds reduced GFP^+^ cell frequency and overall cell viability, indicating that diminished GFP^+^ signals are attributable to cytotoxic effects. Both RGFP966 and mod-RGFP966 induced HIV expression in the primary CD4^+^ T cell latency model at 4- and 24-hour time points, with mod-RGFP966 showing superior efficacy at 4-hour time point (Fig. 3C). To examine whether this altered activity is due to HDCR inhibition, we analyzed histone modification in HIV latency model 2D10 and found that the mod-RGFP966 exhibited stronger activity in enhancing crotonylation with minimal impact on acetylation compared to the parental RGFP966 (Fig. 3D).

To further dissect fluorine’s role in HDCR activity, we leveraged mocetinostat (*37*), a less selective HDACi lacking fluorine on its benzamide ZBG (Fig. 2, D and E), and synthesized a fluorine-modified analog (mod-mocetinostat) by introducing fluorine into this structural motif (Fig. 3E). Mod-mocetinostat exhibited reduced potency compared to mocetinostat in reversing HIV latency at lower concentrations (0.5 to 2 μM) but surpassed the parent compound’s activity at higher concentrations (5 and 10 μM) (Fig. 3F), with no associated cytotoxicity (fig. S2B). These observations indicate that some of the other pathways of HIV latency reversal related to mocetinostat/mod-mocetinostat are involved, possibly because of its dual activity to both HDCR and HDAC. Histone modification analysis in 2D10 cells revealed that mod-mocetinostat induced both crotonylation and acetylation, mirroring the parental compound, although levels were lower than those observed with unmodified mocetinostat when administered at 5 and 10 μM (Fig. 3G). We extended our analysis to a primary CD4^+^ T cell model of HIV latency. Consistent with earlier results, mod-RGFP966 exhibited significantly enhanced latency-reversing potency compared to the parent RGFP966 at all tested doses. Conversely, mod-mocetinostat showed a trend to reduce latency reversal, where at 1 μM, it markedly reduced efficacy relative to its unmodified counterpart (Fig. 3H). Together, these chemical biology studies suggest that fluorine on the benzamide ZBG does not influence HDCR selectivity. Furthermore, its removal in RGFP966 unexpectedly enhances its activity toward inhibiting HDCR and significantly improves its latency-reversing potency.

### Understanding the structural basis of selective HDCR by molecular docking of HDACis against the catalytic pockets of the HDAC3 structure

**Fig. 4.**
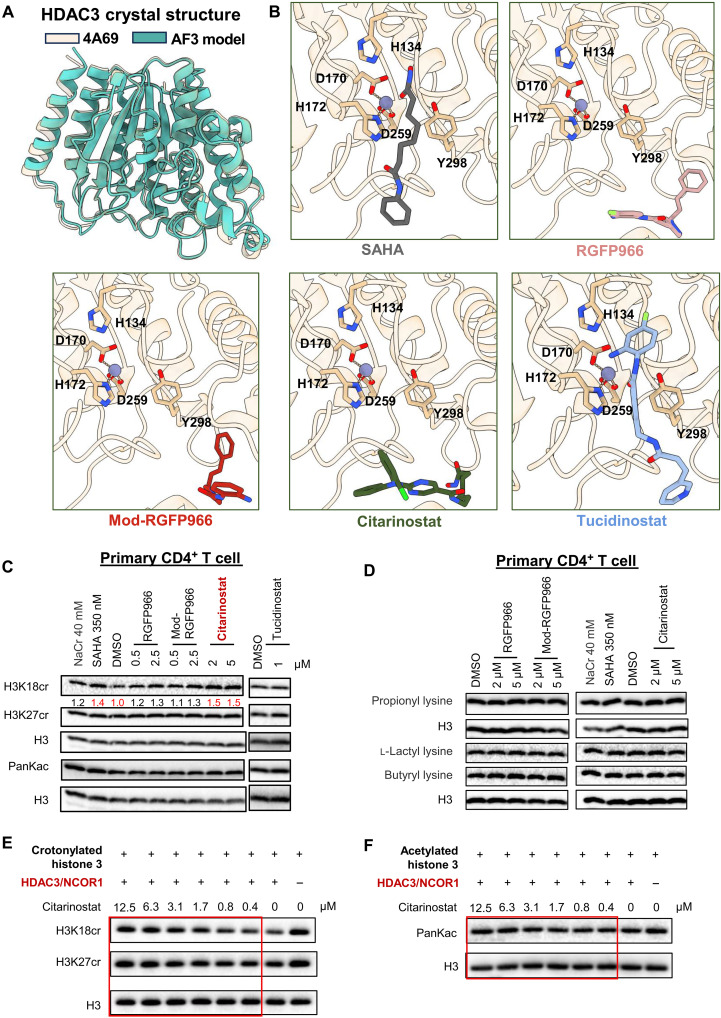
Structural and functional characterization of HDAC3i reveals mechanisms of HDCR modulation. (**A**) A modified version of the HDAC3 crystal structure (PDB: 4A69) with the DAD and IP_4_ removed compared with the AF3 predicted HDAC3 crystal structure. (**B**) Docking the selected HDACis into the enzyme pocket of HDAC3. (**C** and **D**) Primary CD4^+^ T cells were treated with the indicated HDACis for 0.5 hours. Total histone H3 and histone PTMs were detected by Western blot. The relative intensity was shown below with results normalized to DMSO control. HDAC3/NCOR1 (0.3 mg/ml) was incubated with crotonylated histone H3 (**E**) or acetylated histone H3 (**F**) in the presence or absence of different concentrations of citarinostat. Total histone H3 and histone PTMs were detected by Western blot. For (C) and (D), DMSO was used as a negative control. Results are representative of three biological replicate experiments with consistent results.

The results above suggest that HDCRi activity is due to neither the unique benzamide ZBG nor fluorine substitution. To uncover the underlying mechanism, we explored the interaction of HDCRi with HDAC3 through molecular docking studies. Unfortunately, almost all the available HDAC3 crystal structures are within an HDAC3 supercomplex. For example, HDAC3 in complex with the deacetylase-activating domain (DAD) of its corepressor complex revealed an inositol 1,4,5,6-tetrakisphosphate (IP_4_) molecule at the protein-protein interface (*45*). To see whether the removal of these proteins would have a potential effect on the structure of HDAC3 alone, we used AlphaFold3 (AF3) to predict the HDAC3 structure. This structure exhibited a root mean square deviation (RMSD) of 0.68 Å (overall) and a trimmed RMSD of 0.572 Å, closely resembling the experimental HDAC3 structure within the supercomplex (Fig. 4A). For this reason, we used a modified version of the crystal structure [Protein Data Bank (PDB): 4A69], with the DAD and IP_4_ removed, and performed docking studies to predict the potential poses of dual-targeting and selective HDACis (Fig. 4B and fig. S3A). SAHA, which can act as an HDACi and an HDCRi, was observed to dock deep within the ZBG pocket of HDAC3, a pattern also shared by many dual-targeting HDACis tested, including tucidinostat. Unexpectedly and in contrast to SAHA, RGFP966 and mod-RGFP966, both identified as selective HDCRis, docked just outside the catalytic pocket. This observation was intriguing, as it suggested that differences in docking patterns could play a critical role in determining the selectivity between deacetylase and decrotonylase activities with HDAC3 protein (Fig. 4B).

To test this hypothesis and to explore this further, we screened additional HDACis to analyze their docking profiles with HDAC3 (Fig. 4B and fig. S3A). While most compounds docked deep within the catalytic pocket, one compound, citarinostat, docked just outside the pocket, similar to the previously identified selective HDCRi RGFP966 or mod-RGFP966. On the basis of this observation, we hypothesized that citarinostat should also exhibit HDCR selectivity over HDAC activity. When tested in HIV latency models, we found that consistent with our prediction, citarinostat preferentially induced histone crotonylation rather than acetylation in 2D10 cells, thereby confirming its classification as another selective HDCRi candidate (fig. S3B). Conversely, most compounds that docked successfully within the catalytic pocket, such as CUDC-101, quisinostat, and CUDC-907, induced both histone crotonylation and acetylation (fig. S3C). Consequently, these compounds were classified as dual-targeting HDACis. Among these, tucidinostat (*46*) uniquely induced strong histone acetylation with minimal crotonylation, which is opposite to citarinostat, suggesting that it may act as a truly selective HDACi (fig. S3C). Notably, this is the only possible candidate of selective HDACi with minimal HDCRi activity found after screening many currently available HDACis.

To assess the latency reversal activity of selective HDACi and HDCRi, we tested citarinostat and tucidinostat in the 2D10 HIV latency model. Both compounds robustly induced HIV transcription (fig. S3D). Citarinostat, a second-generation, orally bioavailable HDAC6-selective inhibitor bearing a hydroxamic acid ZBG (fig. S3E), typically inhibits HDAC6 to induce α-tubulin hyperacetylation (*39*). However, in 2D10 cells, citarinostat did not increase α-tubulin acetylation within 1 hour posttreatment (fig. S3F), further indicating the HDCR selectivity of citarinostat in the early time point. Notably, dual-targeting HDACis also potently reversed HIV latency (fig. S3G), consistent with previous reports of HDACis functioning as LRAs.

In primary CD4^+^ T cells, HDCRi citarinostat selectively enhanced histone crotonylation over acetylation even within 30 min of treatment (Fig. 4C). In contrast, RGFP966 and mod-RGFP966, weaker LRAs compared to citarinostat (Fig. 3B and fig. S3D), only weakly induced crotonylation at this early time point, with RGFP966 showing minimal activity. However, in contrast to its activity in the Jurkat model of latency, tucidinostat failed to induce either acetylation or crotonylation 30 min posttreatment in the primary CD4^+^ T cell model of latency (Fig. 4C).

Recent discoveries have identified diverse short-chain lysine acylations on histones, including not only acetylation and crotonylation but also butyrylation, propionylation, and l-lactylation. Recognizing the diversity of histone acylation modifications (*20*), we sought to investigate whether the HDCRis we identified above were truly selective for decrotonylase activity over other acylations. Analysis of histone propionylation, lactylation, and butyrylation activity revealed that RGFP966, mod-RGFP966, and citarinostat induced minimal changes in these acylation modifications in both 2D10 cells (fig. S3H) and primary CD4^+^ T cells (Fig. 4D).

Pharmacological approaches in cells are complicated by the potential coexpression of multiple HDACs, some of which have dual deacetylase and decrotonylase activities (*21*). To definitively dissect these enzymatic activities in the context of inhibitors, we require a well-controlled in vitro biochemical assay using purified HDAC3 and defined acylated substrates (acetylated and crotonylated). While numerous commercial kits exist for quantifying classic HDAC deacetylase activity, no equivalent direct assay is available for measuring decrotonylase (HDCR) activity. Therefore, we adapted a published in vitro decrotonylation/deacetylation assay (*47*). This method involves incubating purified HDAC3 with crotonylated or acetylated histone substrates, followed by detection of the remaining acyl mark using site-specific antibodies. Incubation of crotonylated histone H3 with HDAC3/nuclear receptor corepressor 1 (NCOR1) (0.3 mg/ml) reduced signal intensity for H3K18 crotonylation (H3K18cr), H3K27 crotonylation (H3K27cr), and, to a lesser extent, pan-lysine acetylation (panKac) marks, confirming expected enzymatic activity (Fig. 4, E and F). In this system, citarinostat potently inhibited the decrotonylase activity of HDAC3, increasing H3K18cr and H3K27cr levels in a dose-dependent manner (effective from 0.8 μM). In contrast, it only slightly attenuated deacetylase activity at a substantially higher concentration (6.3 μM) (Fig. 4, E and F). We further tested higher enzyme concentrations (HDAC3/NCOR1, 0.45 and 0.6 mg/ml) to amplify the deacetylase signal. Under these conditions, HDAC3/NCOR markedly reduced histone acetylation. Even so, citarinostat’s inhibition of deacetylase activity remained minimal (fig. S4). This differential inhibition profile validates citarinostat’s selectivity for decrotonylation over deacetylation. Collectively, these results suggest that inhibitors targeting sites outside the canonical catalytic pocket of HDAC3 may be key to achieving selective decrotonylase inhibition.

### Citarinostat binds a conserved external site on HDAC3 and HDAC8 to selectively inhibit decrotonylase activity

Citarinostat is a potent biochemical inhibitor of HDAC6 but shows reduced efficacy against the nuclear class I HDACs (HDAC1, HDAC2, HDAC3, and HDAC8) (*48*), all of which reported have dual deacetylase and decrotonylase activities (*21*). To investigate whether citarinostat’s noncanonical binding mode, targeting sites outside the catalytic pocket of HDAC3, is conserved across other class I members, we performed comparative molecular docking analyses against HDAC1, HDAC2, HDAC3, and HDAC8. The pan-inhibitor SAHA, with its well-characterized zinc-chelating binding mode, served as an internal control.

**Fig. 5.**
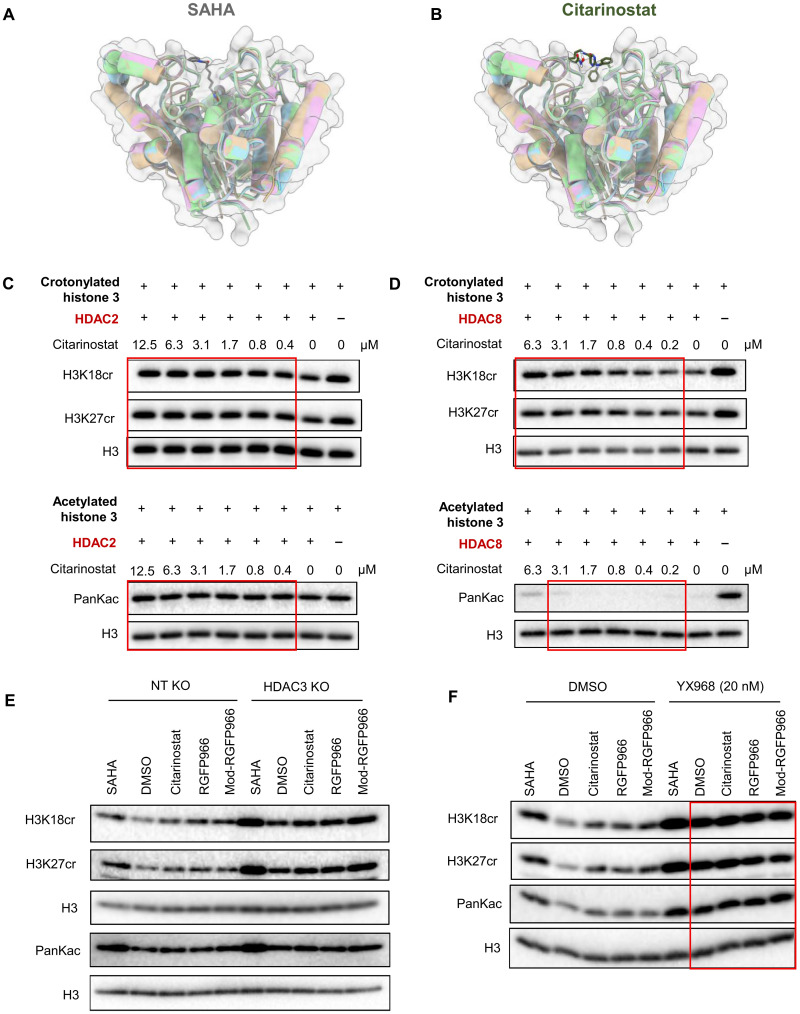
Citarinostat binds a conserved external site on HDAC3 and HDAC8 to selectively inhibit decrotonylase activity. Structural overlay of HDAC1, HDAC2, HDAC3, and HDAC8 bound to SAHA (**A**) and citarinostat (**B**). HDAC1, green; HDAC2, cyan; HDAC3, wheat; HDAC8, pink. HDAC2 (0.05 mg/ml) (**C**) or HDAC8 (0.4 mg/ml) (**D**) were incubated with crotonylated histone H3 or acetylated histone H3 in the presence or absence of different concentrations of citarinostat. Total histone H3 and histone PTMs were detected by Western blot. (**E**) HDAC3 was knocked out in 2D10 cells via CRISPR-Cas9, using a nontargeting guide RNA as the control, and the cells were cultured for 5 days. NT, nontargeting control; KO, knock out. (**F**) In a separate experiment, 2D10 cells were pretreated for 24 hours with DMSO or 20 nM YX968, an HDAC3/8 dual PROTAC. For both experimental series, cells were subsequently treated for 30 min with the following HDACis: DMSO (vehicle), 350 nM SAHA, 0.1 μM citarinostat, 5 μM RGFP966, or 5 μM mod-RGFP966. Histones were then extracted and analyzed by Western blot for specific PTMs and total histone H3 levels.

Our docking protocol reliably reproduced the canonical binding mode: SAHA consistently occupied the catalytic pocket across all four HDACs, engaging in expected hydroxamate-Zn^2+^ coordination and pocket interactions (Fig. 5A). Two-dimensional interaction maps further confirmed this conserved binding pattern, with key shared residues highlighted (fig. S5A). In contrast, citarinostat exhibited a distinct and reproducible binding profile. For all four HDAC isoforms, the top-ranked docking poses localized outside the canonical catalytic pocket, aligning with the external, cap-proximal binding mode we initially observed for HDAC3 (Fig. 5B). Analysis of interaction diagrams revealed a conserved set of surface residues that mediate citarinostat binding across the isoforms (fig. S5B). This consistent outside-pocket placement suggests that citarinostat engages a shared structural feature on the HDAC surface rather than the deep catalytic tunnel, potentially explaining its unique functional selectivity.

To determine whether citarinostat differentially inhibits the dual enzymatic activities of class I HDACs, we extended our in vitro decrotonylation/deacetylation assay to HDAC2 and HDAC8, since our in vitro study showed that HDAC1 had minimal activity in the inhibition of Tat transactivation of HIV in the TZM-bl reporter model (fig. S1A). We first confirmed that both HDACs have the hypothesized dual activity, effectively removing acetyl and crotonyl marks from histone substrates. Subsequent testing with citarinostat revealed a potent and selective inhibition of decrotonylase activity. In HDAC2, this inhibition elevated histone crotonylation levels at concentration above 0.4 μM. Notably, in HDAC8, citarinostat caused a marked dose-dependent increase in H3K18cr and H3K27cr levels at dosages between 0.4 and 6.3 μM. In contrast, citarinostat’s effect on deacetylase activity was markedly weaker for both HDAC isoforms, requiring substantially higher concentrations for only partial attenuation (12.5 μM for HDAC2 and 6.3 μM for HDAC8) (Fig. 5, C and D).

To determine whether HDAC3 is required for citarinostat to induce histone crotonylation, we performed CRISPR-Cas9 knockout of HDAC3 in 2D10 cells. Specific loss of HDAC3 protein, without affecting the HDAC1, HDAC2, or HDAC8 level, was confirmed (fig. S5C). Following HDAC3 knockout, the ability of citarinostat, RGFP966, and mod-RGFP966 to increase histone crotonylation was not completely abolished, producing an effect slightly above the dimethyl sulfoxide (DMSO) control (Fig. 5E). This result indicates that citarinostat can elevate crotonylation through other HDACs in addition to HDAC3.

Given that citarinostat also targets the decrotonylase activity of HDAC8 (Fig. 5D), in addition to HDAC3 (Fig. 4E), we used YX968, an HDAC3/HDAC8 dual proteolysis targeting chimera (PROTAC), to degrade both enzymes (*49*). We first confirmed that 1 to 20 nM YX968 (MedChemExpress) selectively degraded HDAC3 and HDAC8, but not HDAC1 and HDAC2, in 2D10 cells, unless the dosage was higher than 50 nM (fig. S5D). Therefore, we pretreated 2D10 cells with DMSO or 20 nM YX968 for 24 hours. Following drug washout, cells were further treated with selected HDACis for another 30 min. Upon HDAC3/8 degradation, the ability of citarinostat, RGFP966, and mod-RGFP966 to increase histone crotonylation was abolished, with effects comparable to the DMSO control (Fig. 5F). This result indicates that citarinostat elevates crotonylation primarily through the inhibition of HDAC3 and HDAC8 but probably not HDAC2.

### Exploring the unique docking profile to predict selective HDCRi

If the unique docking profile of compounds outside the HDAC3 catalytic pocket is a prerequisite for effective HDCRi, then we should identify other selective HDCRi based on this unique docking profile. To this end, we sought virtual screening of compound libraries with 1101 US Food and Drug Administration (FDA)–approved compounds from the PubChem database. Of these, 743 compounds exhibited binding poses outside the HDAC3 catalytic pocket, suggesting potential selectivity as HDCRi candidates. To refine this selection, we prioritized compounds based on binding affinity scores and interaction patterns. The three highest-scoring compounds, lonafarnib, olaparib, and deferasirox, were chosen for further evaluation. These compounds demonstrated favorable docking energies ranging from −9.9 to −8.0 kcal/mol, indicating strong binding interactions (fig. S6A). The docking analysis revealed that these selected compounds preferentially interacted with residues outside the enzymatic zinc binding pocket (ZBP). Notably, lonafarnib (*50*) and olaparib (*51*) induced HIV transcription from latency, whereas deferasirox (*52*) failed to elicit any reactivation (fig. S6B). Further biochemical analysis revealed that lonafarnib and olaparib increased histone crotonylation in a dose-dependent manner, aligning with their proposed role as HDCRi that block decrotonylation. In contrast, deferasirox barely modulated histone crotonylation; thus, no HIV transcription was enhanced or inhibited (fig. S6C). Notably, neither lonafarnib nor olaparib altered global levels of histone acetylation, butyrylation, propionylation, or l-lactylation (fig. S6C), supporting the hypothesis that compounds docking outside the HDAC3 catalytic pocket lack intrinsic broad-spectrum acylation modulation. Lonafarnib is a farnesyltransferase (FTase) inhibitor, which shows some activity in disrupting latent HIV (*53*, *54*). However, the mechanism of FTase-induced latency reversal is elusive. Our data here found an unexpected activity of FTase inhibitor lonafarnib as a possible selective HDCRi. Collectively, these results further support the premise that the region or structure just outside the HDAC3 catalytic pocket is critical for HDCR activity in HDAC3 protein.

### Amino acids surrounding the pocket sites are critical in maintaining HDCR activity in HDAC3

**Fig. 6.**
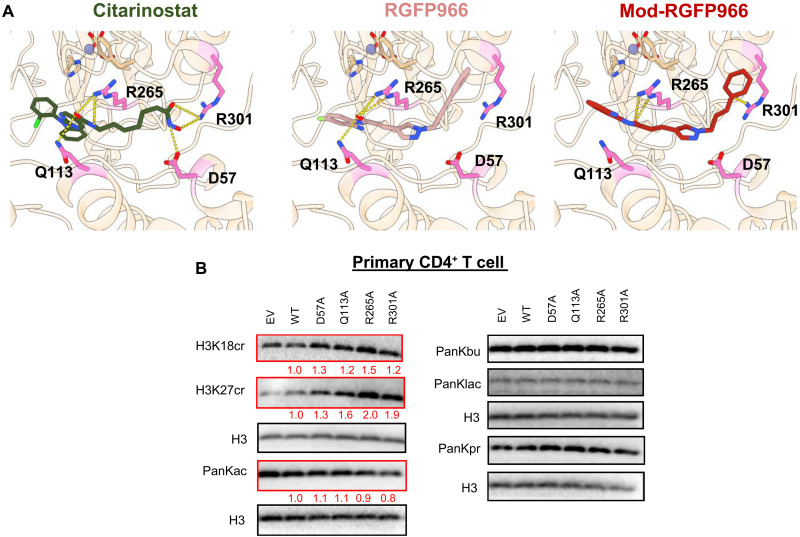
Amino acid mutations surrounding the pocket sites diminish HDCR activity within HDAC3 decrotonylation enzyme. (**A**) Citarinostat, RGFP966, and mod-RGFP966 were docked against the artificial intelligence–generated enzyme pocket of HDAC3. Single-letter abbreviations for the amino acid residues are as follows: D, Asp; Q, Gln; R, Arg. (**B**) Primary CD4^+^ T cells were nucleofected with 1 μg of EV or HDAC3 WT/D57A/Q113A/R265A/R301A. After 5 to 7 days, total histone H3 and histone PTMs were detected by Western blot. The relative intensity was shown below with results normalized to WT HDAC3. PanKbu, butyryllysine; PanKlac, L-lactyllysine; PanKpr, propionyllysine.

Our structural analyses revealed that selective HDCRi preferentially dock adjacent to, but outside, the ZBP of HDAC3. This distinct docking profile suggests that the local structural environment surrounding the catalytic site, rather than the zinc-binding domain itself, plays a critical role in mediating HDCRi selectivity. To map these interactions, we modeled selective HDCRi compounds within the HDAC3 crystal structure, identifying key amino acid residues (D57, Q113, R265, and R301) outside the ZBP that are essential for histone decrotonylase activity (Fig. 6A). To validate their mechanistic role, we generated site-directed HDAC3 mutants (D57A, Q113A, R265A, and R301A) and assessed their decrotonylation activities. Notably, all mutants exhibited a near-complete loss of HDCR activity, thus increasing crotonylation levels compared with WT HDAC3 (Fig. 6B). These HDAC3 mutations had minimal effects on other histone acylation marks compared to WT HDAC3 (e.g., acetylation, l-lactylation, butyrylation, and propionylation), confirming their selective role in decrotonylation, highlighting their essential role in controlling the switch from HDAC to HDCR enzyme activity within HDAC3. These results redefine the structural basis of HDAC3 substrate specificity, emphasizing the importance of peripheral catalytic site architecture in mediating the selective enzymatic activity of HDCR.

### HDCRi selectively induces histone crotonylation, but not acetylation, at the HIV LTR during latency reversal

**Fig. 7.**
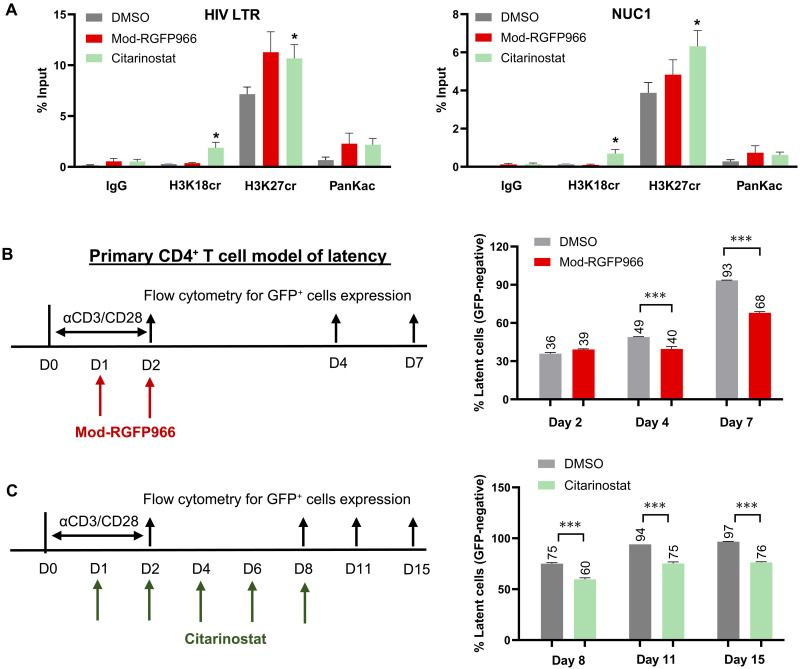
HDAC3-targeted inhibition disrupts histone crotonylation to reactivate latent HIV in latency models. (**A**) 2D10 cells were treated with 0.5 μM mod-RGFP966 and 5 μM citarinostat for 24 hours. Immunoprecipitation was performed using antibodies specific to H3K18cr, H3K27cr, and panKac. Precipitated DNA fragments were amplified using LTR- and NUC1-specific primers and detected by a SYBR green qPCR (*n* = 6). (**B** and **C**) Primary CD4^+^ T cell model of latency was reactivated with anti-CD3/CD28 Dynabeads. After 2 days, activation beads were removed. Cells were further treated with 1.5 μM mod-RGFP966 on day 1 (D1) and day 2 (B) or with 1 μM citarinostat at day 1, 2, 4, 6, and 8 (C) (*n* = 3). Cells were collected, and the expression of GFP cells was analyzed by flow cytometry at the indicated time points. Error bars represent SD; *P < 0.05; ***P < 0.001.

The above data showed that HDCRis disrupt latent HIV by selectively targeting HDCR to induce histone crotonylation, without affecting acetylation. However, these effects were initially assessed at the total histone protein level. To determine whether HDCRi preferentially targets crotonylation surrounding the HIV promoter, we evaluated the histone crotonylation markers H3K18cr and H3K27cr, along with panKac, via chromatin immunoprecipitation (ChIP) assays (*12*). Results revealed that citarinostat significantly elevated H3K18cr and H3K27cr levels at the HIV promoter LTR and nucleosome 1 (Nuc1) regions. Consistent with prior findings, RGFP966 demonstrated a trend toward enhancing H3K27cr; however, the observed increase did not reach statistical significance. Notably, both HDCRi compounds had minimal effect on acetylation near the HIV LTR (Fig. 7A). Together, these findings indicate that selective HDCRi enhances crotonylation at the HIV promoter, highlighting this unique mechanism as a driver of HIV latency reversal.

### Suppression by selective HDCRi may delay HIV reentry into latency in the primary CD4^+^ T cells

If HDCR is critical for establishing HIV latency, then its suppression should prevent or delay viral entry into latency (*55*). Our findings demonstrate that selective HDCRi acts as potent HIV LRAs. We hypothesized that blocking HDCR activity could inhibit proviral transition into latency. To test this, we used a primary CD4^+^ T cell model (*27*), as shown in Fig. 1A, wherein cells were stimulated with anti-CD3/CD28 Dynabeads on day 0. HDCRi treatment commenced on day 1, followed by Dynabeads removal on day 2. HDCRi administration continued for subsequent days. In DMSO-treated controls, GFP-negative cells progressively increased, reflecting natural latency establishment after removal of anti-CD3/CD28, with 93% entering latency by day 7. In contrast, mod-RGFP966 reduced latent infection to 68% (Fig. 7B), while in a separate primary CD4^+^ T cell clone, citarinostat decreased latently infected cells from 97% (DMSO control) to 76% (Fig. 7C). These results demonstrate that disruption of latent HIV at the time of latency establishment by HDCRi may suppress HIV latency reentry.

### Inhibiting HDCR disrupts latent HIV in resting CD4^+^ T cells from PWH

**Fig. 8.**
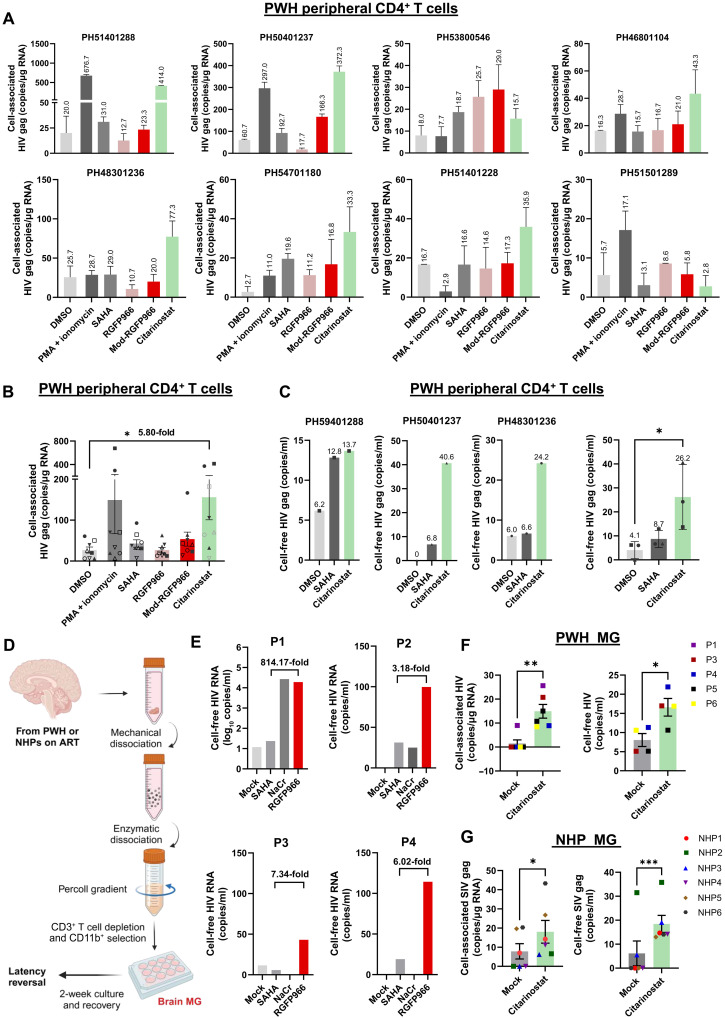
Selective HDCRis disrupt latent HIV in T cells and non–T cells isolated from PWH or NHPs on ART. (**A** and **B**) Peripheral resting CD4^+^ T cells were isolated from eight PWH on ART and treated for 24 hours with the following compounds: PMA (200 ng/mL), ionomycin (2 µM), SAHA (350 nM), RGFP966 (2.5 µM), mod-RGFP966 (2.5 µM), and citarinostat (5 µM). (A) The cell-associated HIV gag levels were measured by droplet digital PCR (ddPCR), with results shown individually for each donor, including triplicate ddPCR data. (B) A combined summary of the triplicate ddPCR results from all donors (*n* = 8) is presented as averages. PMA 200 ng/mL; ionomycin 2 µM; SAHA 350nM; RGFP966 5µM; mod-RGFP966 5µM; citarinostat 5µM. (**C**) Cell-free HIV gag levels in the culture supernatant from three donors treated with 350 nM SAHA and 5 µM citarinostat were measured. (**D**) Brain tissue from NHP or participants with HIV was mechanically dissociated to generate a single-cell suspension, followed by Percoll gradient separation. CD3^+^ T cells were depleted using a CD3 selection kit. Brain MG were isolated from the CD3-negative fraction by CD11b^+^ selection. Brain MG were cultured ex vivo for 1 to 2 weeks to allow recovery and attachment before being used in the LRA study. (**E**) Brain MG from participants with HIV were treated with DMSO (control), 40 mM NaCr, 350 nM SAHA, or 10 μM RGFP966 (*n* = 4). (**F**) In a separate experiment, cells were treated with DMSO or 5 μM citarinostat (*n* = 5). (**G**) Brain MG from NHPs were treated with 5 μM citarinostat or DMSO control (*n* = 6). After 24 hours, the drugs were removed, and cells were cultured for another 6 days. Cells and culture supernatants were harvested for HIV LTR RNA [(E) and (F)] or SIV RNA (G) measurement by ddPCR. A combined summary of the triplicate ddPCR results is presented as averages. Error bars represent SD; *P < 0.05; **P < 0.01; ***P < 0.001.

To further examine whether our previously uncharacterized selective HDCRi could be used in HIV cure studies, we extended our investigation to resting CD4^+^ T cells from PWH on suppressive ART (*n* = 8; Table 1). Phorbol 12-myristate 13-acetate (PMA) and ionomycin, serving as positive controls, induced HIV transcription in six of eight samples, confirming the responsiveness of the model and consistent with our previous observations that while potent in latency reversal, PMA/ionomycin may disrupt latent HIV in all PWH-derived CD4^+^ T cells (*56*). The classical dual-targeting HDACi SAHA induced HIV transcription in three of eight samples, demonstrating limited efficacy as previously reported (*14*). As expected, the selective but weaker HDCRi RGFP966 and mod-RGFP966 induced latency reversal in two to three of eight samples. Notably, citarinostat, the most potent HDCRi we characterized so far, robustly reactivated latent HIV in seven of the eight individuals tested (Fig. 8A and fig. S7A). In most cases, it was even more potent in disrupting latent HIV than PMA/ionomycin. In summary, citarinostat significantly outperformed any other compound in inducing HIV transcription from latency, presenting a remarkable potential for latency reversal in resting CD4^+^ T cells from PWH on ART (Fig. 8B and fig. S7B). In addition, stimulation with citarinostat increased cell-free HIV RNA levels in the culture supernatants (*n* = 3), further validating its potency in latency disruption (Fig. 8C). To our knowledge, this is the only epigenetic LRA that has such superior activity in reversing HIV latency. These results collectively suggest that selectively targeting HDCR can reverse HIV latency, further supporting the unique role of HDCR in regulating HIV latency in resting CD4^+^ T cells.

**Table 1.** Characteristics of HIV-positive participant on ART. PID, participant identification; M, male; F, female.

PID	Gender	Age	Ethnic origin	pVL (copies/ml)	CD4 counts (cells/μl)	ART
51401288	F	59	Black/African American	<20	1365	Biktarvy
50401237	M	43	Hispanic	<20	686	Descovy, Tivicay
53800546	M	63	White	<20	622	Delstrigo
46801104	M	27	White	30	977	Biktarvy
48301236	M	41	Black/African American	<20	773	Biktarvy
54701180	M	29	Black/African American	<20	626	Biktarvy
51401228	F	44	Black/African American	<20	1365	Biktarvy
51501289	F	40	White	<40	1166	Biktarvy

### Selective HDCRi can disrupt latent HIV in non–T cells isolated from PWH or nonhuman primate on ART

The data presented above underscores the pivotal role of HDCRi in reversing HIV latency within T cell reservoirs. However, HIV reservoirs also persist in tissue myeloid cells. For example, we recently characterized a stable HIV reservoir in the brain microglia (MG) (*57*), which may differ from T cell reservoirs regarding epigenetic regulation (*58*). Reversal of HIV latency in both T cell and non–T cell reservoirs is critical for future cure strategies. Using the same approaches as before (*57*), we isolated viable brain MG from PWH or nonhuman primates (NHPs) on ART (Fig. 8D). As shown in Fig. 8E, selective HDCRi RGFP966 was able to disrupt latent HIV to induce HIV transcription in all tested samples (*n* = 4), while both the dual-targeting epigenetic LRA SAHA and NaCr showed limited efficacy, except in the participant 1 (P1) sample treated with NaCr. Furthermore, citarinostat significantly induced HIV transcription from latent infected human brain MG (Fig. 8F). Expanding our investigation in simian immunodeficiency virus (SIV)–infected NHPs on ART (*n* = 3) or after ART interruption (*n* = 3). We found that, similar to human MG, selective HDCRi citarinostat significantly up-regulated SIV transcription in isolated brain MG and increased SIV RNA release from these cells (Fig. 8G and fig. S8, A and B).

Notably, two animals, NHP1 and NHP3 from California National Primate Research Center (CNPRC), had plasma viral load (pVL) between 290 and 400 copies/ml at the time of necropsy, despite continuous ART (table S1). Despite that, cell-associated SIV RNA was undetectable in brain MG, but SIV RNA expression and viral release were induced by citarinostat (Fig. 8G). Animals 4, 5, and 6 from Oregon National Primate Research Center (ONPRC) experienced ART interruption but maintained low plasma viremia for a prolonged time and thus were considered as posttherapy controllers. Nevertheless, SIV transcription in brain MG remained extremely low in these cells, as well as in culture supernatants, but latent SIV was induced by citarinostat (Fig. 8G). Together, these findings underscore the critical and unique role of HDCR in HIV or SIV latency in brain MG in PWH on suppressive ART or NHPs. By targeting diverse cellular reservoirs in both the periphery and the central nervous system (CNS), selective HDCRi inhibition could contribute to developing strategies for better HIV latency reversal.

## DISCUSSION

The establishment and maintenance of HIV latency are governed by complex epigenetic regulations. In this study, we demonstrate that HIV latency is uniquely regulated by histone crotonylation in both T cells and non–T cell reservoirs, such as brain MG, part of the immune-privileged CNS, and a stable HIV myeloid cell reservoir we have recently characterized (*57*). This finding underscores the pivotal role of HDCR in HIV persistence and positions the selective targeting of this pathway with inhibitors such as citarinostat as a potent strategy for latency reversal.

A central contribution of this work is the definitive identification of HDAC3 and HDAC8 as the major HDCR enzymes and the primary cellular targets mediating citarinostat’s procrotonylation effect. This conclusion was established through a stepwise functional dissection of class I HDACs, enzymes known to have HDCR activity (*21*). Initial experiments using HDAC3 mutants defective in deacetylase activity confirmed that HDAC3 suppresses Tat-mediated HIV transcription via its decrotonylase function. In contrast, parallel analysis of HDAC1 did not show a similar suppressive function (fig. S1A). Because of the possibility that other HDACs have dual activity, including HDAC2 and HDAC8, might compensate for the loss of decrotonylation activity during our cellular analyses of HDCR chemical biology in HDAC3 (*59*), we used orthogonal genetic and chemical approaches. CRISPR-Cas9 knockout of HDAC3 only partially reduced citarinostat’s ability to induce histone crotonylation, suggesting compensatory activity from other HDACs. We therefore used the dual PROTAC YX968 to degrade both HDAC3 and HDAC8. This intervention completely abolished citarinostat’s effect, providing compelling evidence that citarinostat elevates crotonylation primarily through HDAC3 and HDAC8, distinguishing their roles from those of HDAC2 in this cellular context.

Although it was not completely unexpected, many currently available HDACis, aside from tucidinostat, demonstrated strong activities in inducing both acetylation and crotonylation within 30 min of exposure. This suggests that previous studies on HIV latency reversal using these HDACis may be partially mediated by the suppression of HDCR as well (*14*, *37*, *60*), a mechanism likely also involved in their activity in cancer (*61*) and acute kidney injury therapy (*62*, *63*). This highlights the potential role in mediating active epigenetic regulation, as observed in our study. It may be worthwhile to revisit earlier studies to more accurately delineate the mechanisms of epigenetic regulation in HIV latency, cancer therapy, or kidney disease treatments. While tucidinostat is selective for HDAC over HDCR and potent in latency reversal in 2D10 cells, it was weak in inducing histone acetylation in primary CD4^+^ T cells when the cells were treated for 30 min. It may warrant further investigation with a longer treatment time. Alternatively, other acylation modifications could be involved in its latency reversal. However, dissecting the specific roles of individual acylation in regulating HIV latency and carcinogenesis remains a challenge, as the same enzymes catalyze many different histone acylations (*20*), which may or may not be controlled by identical enzyme pockets or substructures, as we have shown for HDCR.

A key feature of our investigation was the examination of fluorine substitution in the benzamide ZBG of HDCRis. While fluorine introduction is a common strategy to optimize pharmacological properties, we characterize the unique and unexpected role of fluorine in the activities of the benzamide ring in HDCRi during HIV latency reversal. The incorporation of fluorine in RGFP966, an HDAC3i, was originally aimed at optimizing its pharmacological properties, including enhanced binding affinity to HDAC3, improved potency in suppressing HDAC3 deacetylation activity, and metabolic stability (*43*, *44*). The removal of the fluorine atom from the benzamide ZBG of RGFP966 appears to enhance its efficacy and reduce cellular toxicity in T cells, suggesting that defluorination might increase the potency of HDCRi inhibition and facilitate the reactivation of latent HIV. Furthermore, for mod-mocetinostat, fluorination (creating mod-mocetinostat) reduced its potency to reverse HIV latency at lower concentrations (0.5 to 2 μM). Fluorine typically participates in weak intramolecular interactions, such as C─F⋯H hydrogen bonds, and may influence the conformation of the ZBG. In RGFP966, fluorine likely stabilizes the orientation of the 2-amino-4-fluorophenyl group, optimizing its interaction with the zinc ion and the HDAC3 active site. This finding suggests that removing the fluorine atom from the benzamide ring could shift the selectivity of RGFP966 from HDAC to HDCR, thereby enhancing its potency in disrupting HIV latency. These results may inform the design of more specific and effective HDCRis with improved pharmacological profiles and reduced toxicity.

Our structural analyses revealed a key mechanism underlying HDCRi selectivity. Comparative molecular docking demonstrated that citarinostat exhibits a conserved noncanonical binding mode across HDAC1, HDAC2, HDAC3, and HDAC8. Unlike the pan-inhibitor SAHA, which consistently occupies the ZBP, citarinostat preferentially docks to an external, cap-proximal site, engaging a shared surface feature on these enzymes. The rigid, extended architecture of their ZBG structures could induce conformation changes in the enzyme’s catalytic pocket (*20*), selectively impairing decrotonylation activity without broadly inhibiting other deacylase functions. This model provides a mechanistic framework for rational discovery, which we validated by identifying additional selective HDCRi, such as lonafarnib and olaparib, from FDA-approved libraries using the same docking paradigm. Their predicted external binding and lack of broad-spectrum deacylation activity reinforce the predictive value of targeting this conserved exosite for selective HDCR inhibition.

Although no decrotonylation-specific substructure has been identified in any HDAC, our model posits the existence of a secondary hydrophobic groove or adjacent pocket, ~5 to 7 Å in length, that could accommodate the crotonyl chain (CH_3_─CH═CH─C═O─). Such a site may stabilize the crotonyl group via π-π interactions and include a distinct catalytic residue for hydrolysis, separate from the zinc. Structural validation, such as cocrystallization of HDAC3 with HDCRi or crotonyl lysine, is needed to clarify these binding poses and refine our mechanistic understanding.

Modeling a few selective HDCRis within the HDAC3 crystal structure identified four key residues (D57, Q113, R265, and R301) in the catalytic site’s peripheral architecture. Mutagenesis of these residues abolished HDAC3’s decrotonylase activity, underscoring their essential role as a molecular selectivity filter. These findings redefine the structural basis of HDAC3 enzymatic specificity and offer therapeutic opportunities due to the high potency of the selective HDCRi citarinostat found in this study. A similar approach can likely identify the essential amino acids surrounding, but not inside, the HDAC enzyme that are required for HDCR activity in HDAC8 protein. Further, histone crotonylation dynamics contribute to epigenetic silencing that may maintain HIV latency. By targeting HDAC3’s decrotonylation activity, either through inhibitors mimicking the mutagenesis-induced disruption or modulation of these residues, latent viral reservoirs could be better reactivated, enabling a follow-up immune clearance and advancing strategies for an HIV functional cure. Together, this work advances our understanding of HDCR activity in regulating HIV latency. The chemical biology approaches used here also provide a blueprint for designing next-generation epigenetic inhibitors that target discrete histone-modifying activities with precision, offering therapeutic potential for diseases linked to aberrant crotonylation signaling.

It is challenging to secure a rapid autopsy that allows isolation of viable brain MG (*57*, *64*). A physiologically relevant brain MG model of HIV latency is lacking. Therefore, further mechanistic investigation of HDCRi activity remains limited. Notably, the selective HDCRi RGFP966 can penetrate the brain (*65*). Our data suggest that selective inhibition of HDAC3 decrotonylase activity by RGFP966 or citarinostat may reactivate latent HIV across multiple tissue compartments, including the CNS, thereby enhancing HIV cure strategies.

Future efforts to design a HDCRi will leverage advanced machine learning, using models like MolGPT (*66*), a transformer-decoder framework that generates diverse molecular structures by learning chemical patterns from simplified molecular input line entry system (SMILES) representations. This approach will be paired with dynamic structural constraints to ensure synthetic feasibility. By integrating expansive chemical libraries and predictive models for potency and selectivity, these strategies aim to streamline the identification of promising candidates. This direction holds substantial potential to accelerate the development of targeted epigenetic therapies, addressing critical gaps in HDCR-related disease treatment.

In summary, our findings highlight the unique role of HDCR in regulating HIV latency in both T cells and the brain MG. The discovery of unique HDCR activity in HDAC2, HDAC3, and HDAC8, selective HDCRi, and amino acids essential for HDCR surrounding the ZBP at class I HDAC, i.e., HDAC3, provides us with an opportunity to unravel the molecular mechanism of HDCR in regulating HIV transcription and latency. Continued exploration of HDCR-specific inhibitors will help better understand the molecular mechanism of HDCR and yield a distinct class of specific and potent epigenetic compounds to enhance current HIV cure strategies by disrupting the extremely stable latent reservoirs.

## MATERIALS AND METHODS

### Primary resting CD4^+^ T cell isolation and culture

Leukocytes were obtained from eight individuals with HIV on ART for at least 3 years, all with undetectable HIV RNA in plasma (Table 1). Resting CD4^+^ T cells were isolated using a custom resting CD4^+^ T cell isolation kit (STEMCELL Technologies) and cultured in RPMI R10 [10% fetal bovine serum (FBS; Avantor), 1% penicillin-streptomycin (Gibco), 10 mM Hepes (Gibco), 2 mM l-glutamine (Gibco), and 1 mM sodium pyruvate (Gibco), supplemented with interleukin-2 (IL-2; 40 U/ml; PeproTech)]. All participants provided informed consent, and the study was approved by the University of North Carolina, Chapel Hill Institutional Review Board (IRB).

### Last Gift rapid research autopsy program

Six participants with HIV included in this study were enrolled from the Last Gift rapid research autopsy program, which recruits altruistic PWH with a terminal illness who are well suppressed on ART for close perimortem follow-up (Table 2). The inclusion criteria for this study were confirmed HIV diagnosis, a life expectancy of less than 6 months, ongoing suppressive ART, and no history of CNS malignancy or immune checkpoint inhibitor chemotherapy. The rapid autopsy tissues were shipped overnight to the UNC lab in medium containing antibiotics and ART for isolation of immune cells as described previously (*57*). The study was approved by the University of California at San Diego (UCSD) IRB. Rapid research autopsies were performed within 6 hours after PWH death, following established protocol (*57*, *64*).

**Table 2.** Clinical demographics of “Last Gift” cohort. TF, transfeminine; FTC, emtricitabine; TAF, tenofovir alafenamide; DTG, dolutegravir; BIC, bictegravir; ABC, abacavir; 3TC, lamivudine; MAI, *Mycobacterium avium*-*intracellulare*; ESRD, end-stage renal disease.

ID	Gender	Age of death	pVL (copies/ml)	CD4 counts (cells/μl)	ART	Clinical diagnoses
P1	TF	72	<30	157	FTC/TAF/DTG	Hepatic cancer
P2	M	48	<30	87	FTC/TAF/BIC	Metastatic anal cancer, Kaposi sarcoma
P3	M	64	<30	162	FTC/TAF/DTG	Esophageal, hepatic cancer
P4	M	32	<30	45	FTC/TAF/DTG	Disseminated multidrug-resistant MAI, ESRD, cardiac arrest 2/2 hyperkalemia
P5	M	44	<30	467	FTC/TAF/BIC	Amyotrophic lateral sclerosis
P6	M	69	<30	287	ABC/DTG/3TC	Metastatic colon cancer

### Animal studies

Rhesus macaques (*Macaca mulatta*) were housed at the ONPRC or CNPRC (table S1). The care and use of NHPs were approved and regulated by the individual Institutional Animal Care and Use Committee. Both animal care facilities are accredited by the US Department of Agriculture and the Association for Assessment and Accreditation of Laboratory Animal Care International. NHPs from ONPRC were intravenously inoculated with 5000 median tissue culture infectious dose (TCID_50_) SIVmac239M and treated with ART [tenofovir disoproxil (5.1 mg/kg per day), emtricitabine (40 mg/kg per day), and dolutegravir (2.5 mg/kg per day) in a solution containing 15% (v/v) kleptose at pH 4.2] beginning 9 days postinfection for a period of >60 weeks before ART interruption >300 days before brain tissue collection. At the time of necropsy, these animals were considered posttherapy controllers with SIV pVLs of <600 copies/ml. NHPs from CNPRC were intravenously infected with 1000 TCID_50_ of SIVmac251. ART (tenofovir, dolutegravir, and emtricitabine) started at week 6 postinfection and was maintained for 14 weeks before brain collection. At the time of necropsy, NHPs from CNPRC had a SIV pVL of <400 copies/ml. Brain tissues from both centers were collected after necropsy and shipped to the UNC HIV Cure Center via overnight special delivery in medium with antibiotics and ART. CNS cells were then immediately isolated from these tissues for latency reversal studies.

### HIV infection in primary CD4^+^ T cells

A primary CD4^+^ T cell infection model was established using cells from donors without HIV (obtained from the University of California, Los Angeles Center for AIDS Research). Briefly, CD4^+^ T cells were activated with anti-CD3/CD28 Dynabeads (Gibco) in RPMI R10 medium supplemented with IL-2 (100 U/ml) for 2 days. Ten million activated cells were then infected with HIV-1 SF162 by centrifugation at 1200*g* for 2 hours at 37°C. Following centrifugation, cells were plated and treated with or without 40 mM sodium crotonate (NaCr) in RPMI R10 supplemented with IL-2 (40 U/ml). One day later, the virus was washed off, the medium was replaced, and NaCr was resupplemented. Cells were harvested for RT-qPCR targeting the HIV LTR at 1, 4, 6, and 8 days postinfection. In addition, p24 enzyme-linked immunosorbent assay was performed in the supernatants 8 days postinfection.

### HIV latency models

2D10 cell model of HIV latency, which carries a lentiviral vector that expresses Tat with H13L mutation and Rev in cis, along with a destabilized enhanced GFP (d2EGFP) replacing Nef (*67*), was provided by J. Karn. 2D10 cells were cultured in RPMI R10 in a 37°C incubator with 5% CO_2_. A primary CD4^+^ T cell model of latency was also used, as previously described (*27*–*29*). Briefly, CD4^+^ T cells obtained from HIV seronegative individuals were infected with pNL4.3Δ6-dreGFP virus. This virus was produced by cotransfecting 293T cells with the pNL43Δ6-dreGFP plasmid (provided by R. Siliciano, Johns Hopkins University) and the packaging plasmids psPAX2 (Addgene) and pMD2.G (VSV-G, Addgene) using Lipofectamine 3000 (Thermo Fisher Scientific). The construct carries premature stop codons in all viral genes except *tat* and *rev*, and a destabilized eGFP gene within the envelope reading frame (*68*). GFP^+^ cells were sorted and subsequently cocultured with H80 feeder cells to facilitate the establishment of HIV latency. Last, latently infected GFP-negative CD4^+^ T cells were sorted and maintained as the primary CD4^+^ T cell model of HIV latency. To reactivate the primary CD4^+^ T cell model of HIV latency, cells were stimulated with anti-CD3/CD28 Dynabeads in RPMI R10 medium supplemented with IL-2 (100 U/ml). After 48 hours of activation, Dynabeads were removed, and the medium was replaced every 2 to 3 days with fresh RPMI R10 containing IL-2 (40 U/ml), maintaining a cell density of 10^6^ cells/ml. Viral gene expression was assessed by measuring GFP expression using flow cytometry.

### HIV gene expression by RT-qPCR analysis or flow cytometry

Total RNA was extracted from the cells using the RNeasy mini kit (QIAGEN) and subsequently treated with deoxyribonuclease I (DNase I; Invitrogen) to remove genomic DNA. First-strand cDNA was synthesized using SuperScript III (Invitrogen) and random primers (Invitrogen). RT-qPCR (TaqMan, Applied Biosystems Inc.) was conducted on an ABI QuantStudio 5 system using primers and probe sets targeting the long LTR or *gag* region of HIV. The succinate dehydrogenase complex flavoprotein subunit A (*SDHA*) primers/probe set (Applied Biosystems Inc.) served as an internal control. HIV transcription was also quantified through GFP expression measured by flow cytometry, with data analyzed using FlowJo software (version X10.9.0). Cell viability was assessed during flow cytometry using live/dead dye (Invitrogen).

### HIV p24 flow cytometry assay

Following NaCr treatment, the frequency of p24-producing cells was quantified via intracellular p24 staining using the KC57–fluorescein isothiocyanate (FITC) antibody (Beckman Colter), as previously described (*69*). Briefly, cells were fixed and permeabilized with Cytofix/Cytoperm solution (BD Biosciences) for 20 min at 4°C. After fixation, cells were washed with Perm/Wash buffer (BD Biosciences) and stained with a 1:40 dilution of the anti-p24 KC57-FITC antibody in 100 μl of Perm/Wash buffer for 30 min at 4°C. Following wash with Perm/Wash buffer, stained cells were analyzed by flow cytometry.

### Luciferase assay

TZM-bl luciferase reporter cells were obtained from the National Institutes of Health HIV Reagent Repository and cultured in Dulbecco’s modified Eagle’s medium (DMEM) D10 (10% FBS; 1% penicillin-streptomycin, 10 mM Hepes, 2 mM l-glutamine, and 1 mM sodium pyruvate). Cells were seeded in a black 96-well plate and incubated overnight to allow cell attachment. The following day, the cells were transfected with 0.03 μg of pcDNA3.1 empty vector (EV), Tat, or HDAC3 WT HDAC3, mutated HDAC3 (HDAC3-VRPP or HDAC3-Y298H), or Tat in combination with WT or mutated HDAC3 using Lipofectamine 3000 Transfection Reagent (Invitrogen). After 48 hours, Tat-driven HIV transactivation was measured using a luciferase assay (Promega). Briefly, cells were washed with phosphate-buffered saline (PBS) and lysed with 20 μl of Reporter Lysis Buffer. The lysates were then mixed with 100 μl of Luciferase Assay Reagent, and the luminescence was measured. The results were normalized to the EV control.

### Western blot

Total cell protein was extracted using radioimmunoprecipitation assay lysis buffer (Sigma-Aldrich) containing a 1× protease and phosphatase inhibitor cocktail (Cell Signaling Technology). Protein expression was assessed using the following antibodies: anti–NF-κB2 p100/p52 (Cell Signaling Technology), anti-ENL (Cell Signaling Technology), anti-CDYL (Gene Tex), anti–β-actin (Cell Signaling Technology), anti–α-tubulin (Cell Signaling Technology), anti–acetyl–α-tubulin (Cell Signaling Technology), anti-HDAC1 (Cell Signaling Technology), anti-HDAC2 (Cell Signaling Technology), anti-HDAC3 (Cell Signaling Technology), and anti-HDAC8 (Cell Signaling Technology). For histone proteins, compounds (see table S2) were added to culture medium for the indicated periods, and total histones were extracted from the cells using the EpiQuik Total Histone Extraction Kit (EpigenTek). Histone modifications were analyzed using the following antibodies: H3K18cr (PTM BIO), H3K27cr (PTM BIO), anti-panKac (i.e., anti–histone H3 acetyl K9 + K14 + K18 + K23 + K27, Abcam), anti–pan–lactyl lysine (PTM BIO), anti–pan–butyryl lysine (PTM BIO), anti–pan–propionyl lysine (PTM BIO), and anti–total histone H3 (Cell Signaling Technology).

### Molecular docking of HDACis against the crystal structure of HDAC3

We used AF3 to predict the effect of the removal of the various corepressors, DAD and IP_4_, found in the protein structure (PDB: 4A69), from HDAC3. After structural alignment between the crystal structure and the predicted sequence, we validated that the crystal structure could be used for molecular docking. The HDAC3 crystal structure (PDB: 4A69) was then modified as follows: We removed the DAD corepressor, IP_4_, and acetate from the structure. The protein was prepared using the Protein Preparation Wizard in Maestro v13.3 (Schrodinger). LigPrep generated the molecular conformers and protonation states from the ligand SMILES strings. The box was centered on residue Y298 next to the Zn^2+^-binding site. Molecular docking was then carried out using Schrodinger’s Glide SP (standard precision), generating up to five poses for each molecule. The top-scoring molecules are used in this analysis. The analysis and molecular visualization were carried out with ChimeraX (www.rbvi.ucsf.edu/chimerax).

### In vitro decrotonylase/deacetylase activity assay

The decrotonylase and acetylase activity assays were performed following an established protocol (*47*). Briefly, 100 μM crotonyl-CoA (Sigma-Aldrich) or acetyl-CoA (Roche) and histone H3 (Active Motif) were incubated with the p300 catalytic domain (Enzo Life Sciences) for 2 hours at 30°C in a thermocycler. Reactions were terminated by heating at 65°C for 5 min. The resulting crotonylated or acetylated histone substrates were then aliquoted into PCR tubes. To assess the effect of citarinostat on decrotonylase or deacetylase activity, the histone substrates were treated with HDAC3/NCOR1 (Enzo Life Sciences), HDAC2 (Abcam), or HDAC8 (Active Motif) in the presence or absence of the inhibitor citarinostat. The reactions were incubated for 2 hours at 30°C in a thermocycler. Control reactions were included: a blank (no HDAC or inhibitor) and an enzyme control (HDAC added without inhibitor). Following incubation, NuPAGE LDS Sample Buffer (4×) (Invitrogen) was added to each reaction, followed by a 5-min incubation at 95°C. Samples were then subjected to Western blot analysis. Histone modifications were detected using antibodies specific to H3K18cr, H3K27cr, panKac, and total histone H3.

### Molecular docking of citarinostat and SAHA against HDAC1/2/3/8

Structural models of human HDAC1, HDAC2, HDAC3, and HDAC8 were generated using AF3 (*70*, *71*) and were subsequently prepared for docking by adding polar hydrogens. Partial charges were assigned, and proteins were converted to the PDBQT format using AutoDockTools. Ligand structures for SAHA and citarinostat were generated from SMILES strings, energy minimized, and prepared with appropriate protonation states before docking. Molecular docking was performed using AutoDock Vina (*72*), which uses an efficient stochastic global search algorithm coupled with an empirical scoring function. Docking grids were centered on the conserved catalytic region adjacent to the zinc-binding site, encompassing the canonical HDAC active-site tunnel as well as the surrounding cap and outer pocket regions. Grid dimensions were selected to allow sufficient conformational sampling of both pocket-bound and extended binding modes. For each ligand-protein pair, multiple binding poses were generated, and the top-ranked pose based on predicted binding affinity was selected for further analysis.

Protein-ligand interactions were analyzed using LigPlot+ (*73*), which provides two-dimensional schematic representations of hydrogen bonds and hydrophobic contacts. These interaction maps were used to identify conserved and isoform-specific residues involved in ligand recognition across HDAC1/2/3/8. Three-dimensional visualization and structural inspection were carried out using UCSF ChimeraX (*74*).

### CRISPR-Cas9 gene knockout

Protospacer sequences targeting *HDAC3* and a nontargeting control were designed by Integrated DNA Technologies (IDT; sequences in table S2). CRISPR-Cas9 ribonucleoprotein (RNP) complexes were assembled by annealing CRISPR RNA and trans-activating CRISPR RNA (IDT) at a 1:1 molar ratio (final concentration, 100 μM) in duplex buffer (IDT) with heating to 95°C, followed by gradual cooling to 25°C. Fresh RNP complexes were prepared for each nucleofection by incubating 49.6 pmol of Alt-R S.p. Cas9 nuclease (62 μM; IDT) with 100 pmol of the annealed guide RNA duplex and 0.8 μl of poly-l-glutamic acid (15 kDa, 100 mg/ml; Alamanda Polymers). For nucleofection, 2D10 cells were pelleted (90*g* for 10 min), washed with PBS, and resuspended in SE Cell Line Nucleofector Solution (Lonza) at 1 × 10^6^ cells per 20 μl. The cell suspension was combined with the RNP complex and transfected using a 4D-Nucleofector device (Lonza) with the CL-120 program. Cells were immediately resuspended in prewarmed R10 medium and cultured at 37°C. Five days postnucleofection, cells were treated for 30 min with DMSO (vehicle), 350 nM SAHA, 0.1 μM citarinostat, 5 μM RGFP966, or 5 μM mod-RGFP966. Histones were then extracted and analyzed by Western blot for specific posttranslational modifications (PTMs) and total histone H3.

### Virtual screening of FDA-approved compounds

To investigate the docking behavior of selective HDCRi within the HDAC3 protein, we first generated a high-confidence structural model of HDAC3. The amino acid sequence of human HDAC3 (UniProt ID: O15379) was retrieved from the UniProt database. The three-dimensional structure was then predicted using the AF3 server (*75*) (https://alphafoldserver.com/). The resulting model was evaluated for structural integrity and used as the receptor for subsequent virtual screening experiments. To identify potential HDCRi candidates, we conducted an in silico screening of 1101 FDA-approved compounds obtained from the PubChem database (https://pubchem.ncbi.nlm.nih.gov/). The molecular structures were downloaded in SMILES format and converted to PDBQT format using Open Babel (Obabel), ensuring compatibility with the docking software. Then, docking simulations were performed using AutoDock Vina, using a search grid (dimensions, 54 Å by 54 Å by 54 Å) centered at coordinates (32.11, −48.947, 33.303) to encompass the entire HDAC3 catalytic site. Default docking parameters were used, including the genetic algorithm (GA) with the following settings: number of GA runs, 10; population size, 150; maximum number of evaluations, 2,500,000; maximum number of generations, 27,000. Each compound was subjected to 20 docking poses to ensure a comprehensive sampling of potential binding interactions. The protein-ligand interaction profiles were visualized and analyzed using ChimeraX to assess binding orientations relative to the enzymatic zinc-binding pocket.

### Plasmid transfection by nucleofection

A plasmid encoding WT FLAG-tagged HDAC3 was obtained from Addgene, and site-directed mutants (D57A, Q113A, R265A, and R301A) were generated by GenScript. For nucleofection, 2D10 cells were pelleted (90*g* for 10 min), washed with PBS, and resuspended in SE Cell Line Nucleofector Solution at a density of 2 × 10^6^ cells per 100 μl. Cells were transfected with 1 μg of either EV control or HDAC3 plasmid using a 4D-Nucleofector device (Lonza) with program CK-116. Following transfection, cells were immediately resuspended in prewarmed RPMI R10 medium and cultured at 37°C. Histones were extracted 5 to 7 days posttransfection and analyzed by Western blot for relevant histone PTMs and total histone H3.

### ChIP assays

ChIP assays were performed as previously described (*76*) with modifications. 2D10 cells were treated with selective HDCRi or DMSO control for 24 hours. Five million cells were cross-linked with 1% formaldehyde (Sigma-Aldrich) by rotation for 10 min at room temperature. To quench cross-linking, 125 mM glycine was added and mixed by inversion. Cells were then washed with PBS (Gibco), and cell pellets were flash frozen in liquid nitrogen and stored at −80°C. Nuclei were isolated by resuspending the cell pellet in 10 ml of rinse buffer #1 [50 mM Hepes (pH 8.0), 140 mM NaCl, 1 mM EDTA, 10% glycerol, 0.5% NP-40, and 0.25% Triton X-100], incubating on ice for 10 min, and centrifuging at 1200*g* for 5 min at 4°C. The pellet was resuspended in 10 ml of rinse buffer #2 [10 mM tris (pH 8.0), 1 mM EDTA, 0.5 mM EGTA, and 200 mM NaCl] and centrifuged again. To remove residual salts, 5 ml of shearing buffer [0.1% SDS, 1 mM EDTA (pH 8.0), and 10 mM tris-HCl (pH 8.0)] was carefully added, and the sample was spun at 1200*g* for 3 min at 4°C, with the wash step repeated. The isolated nuclei were resuspended in 90 μl of shearing buffer (with 1× protease inhibitor) and transferred to glass microtubes (Covaris). Next, 10 μl of nanodroplet cavitation reagents (MegaShear, Triangle Biotechnology) were added to the microtubes. Samples were sonicated in a Covaris E110 instrument at 4°C for 7.5 min. After sonication, the chromatin was immunoprecipitated using antibodies against normal rabbit immunoglobulin G (IgG) (Cell Signaling Technology), H3K18cr, H3K27cr, and panKac. Coimmunoprecipitated DNA was recovered using the ChIP Clean & Concentration Kit (Zymo Research) and analyzed by SYBR Green qPCR (Applied Biosystems Inc.) with primers targeting the HIV LTR and Nuc1 regions. Data were presented relative to the input control.

### Isolation and culturing of brain MG from PWH and NHPs

We isolated the brain MG from PWH and NHPs following our published protocol (*57*). Briefly, fresh brain tissue was mechanically dissociated in 1× Hanks’ balanced salt solution (Gibco) containing DNase I. The released cells were transferred to a 50-ml conical tube. Myelin debris was removed using a 35% Percoll density gradient (*77*) to generate a single-cell suspension of CNS cells. The isolated cells were resuspended in 5 ml of ACK lysis buffer (Gibco) and incubated at 37°C for 10 min to remove red blood cells. After centrifugation, immune cells were then depleted from the other CNS cell population using CD3^+^ T cell selection. For human samples, the EasySep Release Human CD3 Positive Selection Kit (STEMCELL Technologies) was used. For NHP samples, the CD3 MicroBead Kit for NHPs (Miltenyi Biotec) was applied. Last, brain MG were purified from the CD3-depleted CNS cell population. For human samples, CD11b MicroBeads, human and mouse (Miltenyi Biotec) were used, while for NHP samples, CD11b MicroBeads, NHP (Miltenyi Biotec) were used. Purified brain MG were cultured in DMEM/F12 supplemented with human or NHP macrophage colony-stimulating factor for 1 to 2 weeks before treatment with LRAs for latency reversal. Brain MG were treated with LRAs or DMSO control. After 24 hours, the drugs were removed, and the cells were cultured for another 6 days. Culture supernatants were harvested for HIV RNA or SIV RNA measurement by RT–droplet digital PCR (ddPCR).

### Cell-associated and cell-free HIV or SIV RNA quantified by ddPCR

Viral RNA was extracted from PWH-derived resting CD4^+^ T cells and brain MG using RNeasy mini kit (QIAGEN), while cell-free RNA was isolated from culture supernatants with QIAamp viral RNA mini kit (QIAGEN). Total RNA was then treated with DNase I to remove genomic DNA, and cDNA was synthesized using the SuperScript IV First-Strand Synthesis System (Invitrogen). HIV RNA was quantified by RT-ddPCR using two sets of primers/probes targeting HIV gag (*78*) or LTR (*79*) regions, while SIV RNA was quantified using two sets of primers/probes targeting the gag region (table S2). PCR cycling conditions were as follows: 95°C for 10 min, followed by 45 cycles of 30 s at 94°C, 60 s at 57°C, and a final droplet cure step at 98°C for 10 min. Droplets were analyzed using QuantaSoft in absolute quantification mode.

### Statistical analysis

Statistical analyses were performed with GraphPad Prism 10 software. In all graphs, the error bars represent the SD and are only shown for experiments with *n* = 3 or greater, as indicated. Comparisons between control and sample datapoints were made using either a two-tailed unpaired Student’s *t* test or two-way analysis of variance (ANOVA) with multiple-comparison analyses or other statistical methods as specified in the figure legends, with the confidence limit for significance set at 0.05 or less.
