# Supplementary material for: Histone decrotonylation plays a distinct role in HIV latency
Source: Sci Adv. 2026 Apr 10;12(15):eaec0149. doi: 10.1126/sciadv.aec0149 (PMC13068052; doi:10.1126/sciadv.aec0149)
Supplement: Supplementary file 1 — Figs. S1 to S8 Tables S1 and S2 [file sciadv.aec0149_sm.pdf]

Science Advances
AAAS

# Supplementary Materials for

## Histone decrotonylation plays a distinct role in HIV latency

Xiaoyi Li *et al.*

Corresponding author: Guochun Jiang, guochun_jiang@med.unc.edu

*Sci. Adv.* **12**, eaec0149 (2026)
DOI: 10.1126/sciadv.aec0149

**This PDF file includes:**

Figs. S1 to S8
Tables S1 and S2

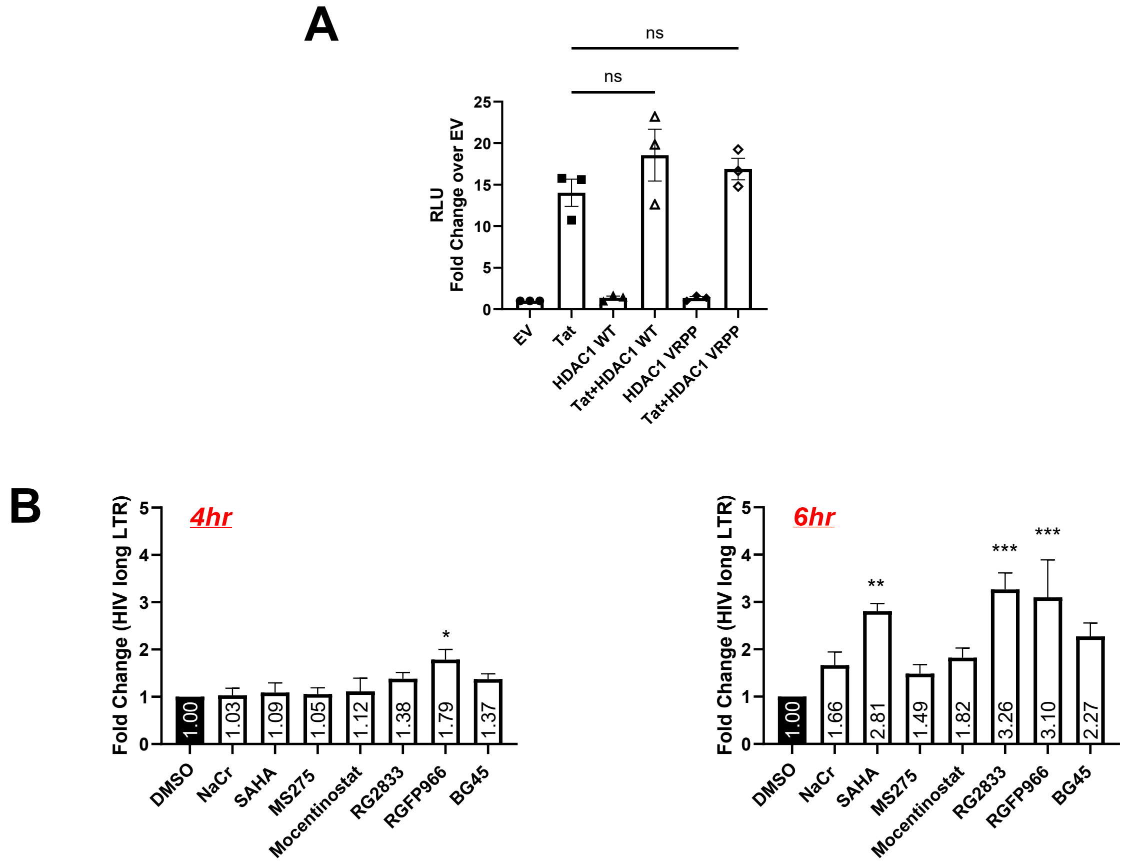


**Fig. S1. HIV transcription of 2D10 cells after treatment with histone deacetylase inhibitors (HDACi).**

(**A**) TZM-bl luciferase reporter cells were transfected with empty vector (EV), Tat, wildtype (WT) HDAC1, or mutant HDAC1 (HDAC1-VRPP), or Tat in combination with WT or mutant HDAC1 for 2 days (n = 3). (**B**) 2D10 cells were treated with indicated HDACi for 4 and 6 hours. HIV transcription levels were measured by RT-qPCR targeting the HIV-1 LTR, with results normalized to DMSO control (n = 5).

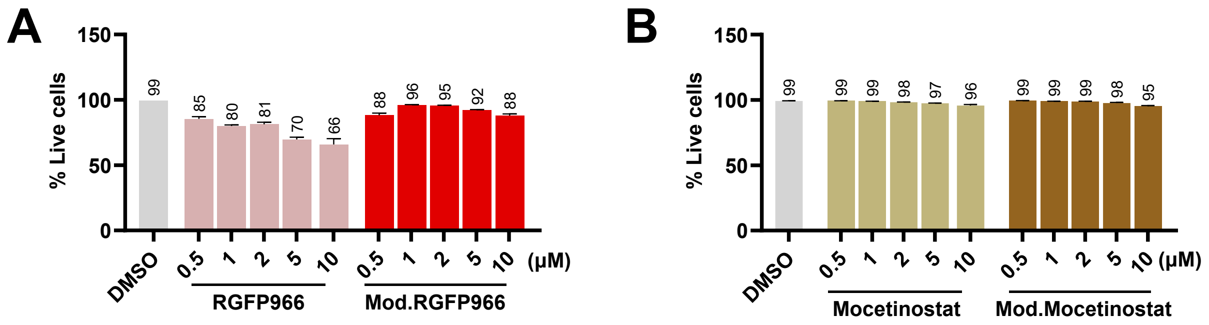


**Fig. S2. Cellular viability of CD4+ T cells after treatment with histone deacetylase inhibitors (HDACi).**

The primary $CD4^+$ T cell model of latency was treated with the indicated selective HDACi for 24 hours, and the percentage of live cells was measured by flow cytometry (n=3).

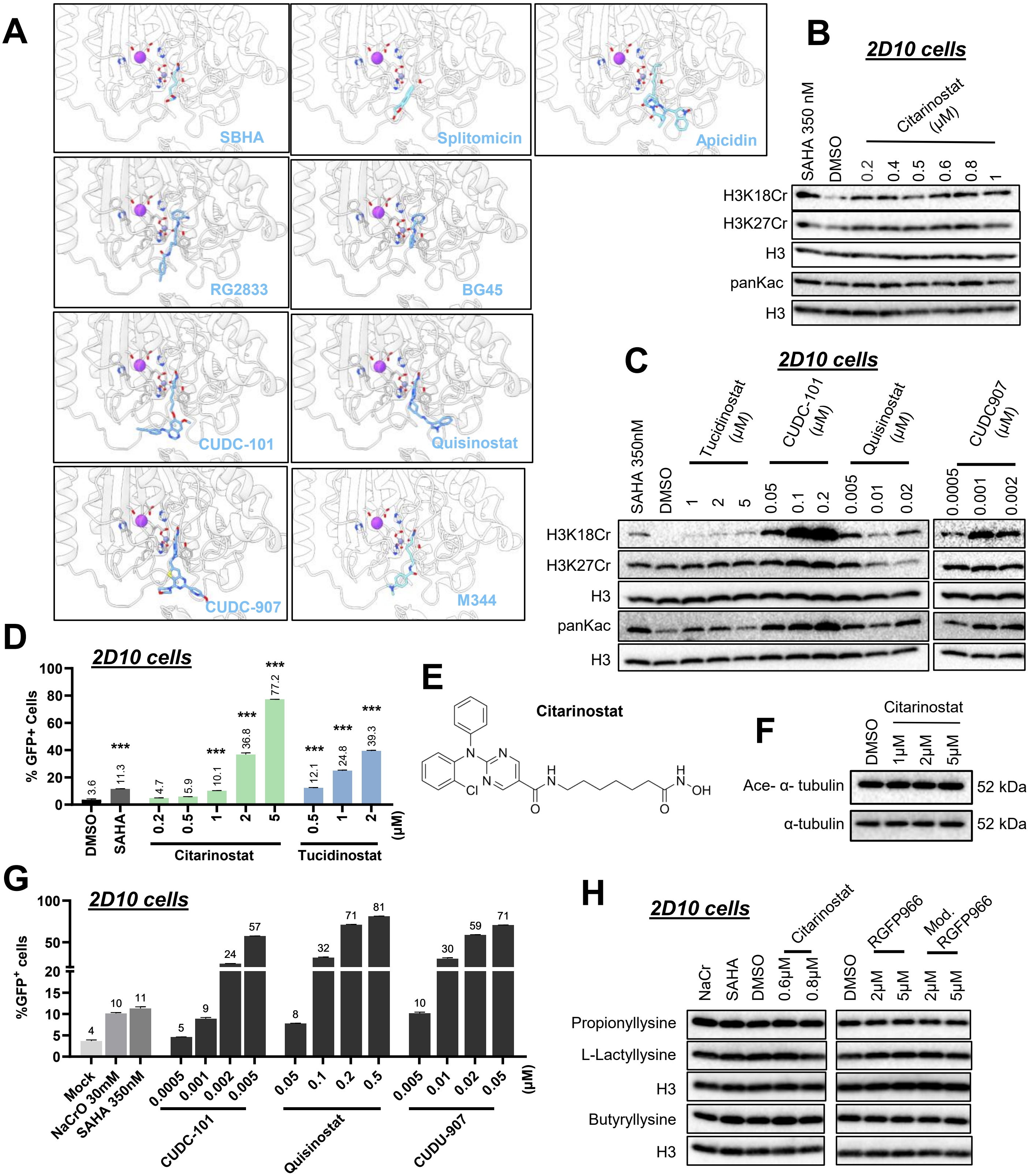


**Fig. S3. Characterization of selective HDACi predicted to dock outside the canonical $Zn^{2+}$ catalytic pocket.**

(**A**) Docking poses of selected HDACi in an AI-modeled HDAC3 structure. (**B, C**) 2D10 cells were treated for 30 min with indicated concentrations of citarinostat (**B**) or other HDACi (**C**); histones were analyzed by western blot. (**D**) GFP expression in 2D10 cells treated with citarinostat or tucidinostat for 24 h, measured by flow cytometry (n=5). (**E**) Chemical structure of citarinostat. (**F**) 2D10 cells treated with citarinostat for 1 h; hyperacetylation and total α-tubulin were assessed by western blot. (G) Percentage of GFP-positive cells after 24 h HDACi treatment, measured by flow cytometry (n=3). (**H**) 2D10 cells treated with HDACi for 30 min; histones were analyzed by western blot. DMSO served as vehicle control in B, C, H. Data are representative of three independent experiments. P values in (**D**) determined by one-way ANOVA with multiple comparisons.

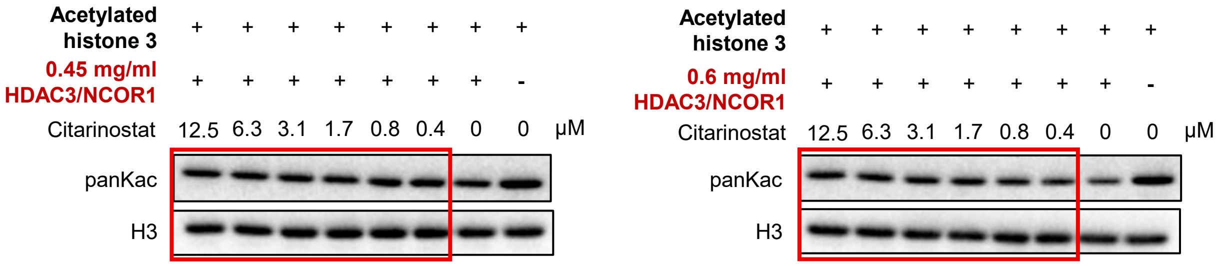


**Fig. S4. Citarinostat's inhibition of deacetylase activity of HDAC3 is minimal.**

Acetylated histone H3 was incubated with HDAC3/NCOR1 (0.45 or 0.6 mg/mL) in the presence or absence of the indicated concentrations of citarinostat. For both assays, total histone H3 and relevant histone modifications were detected by western blot.

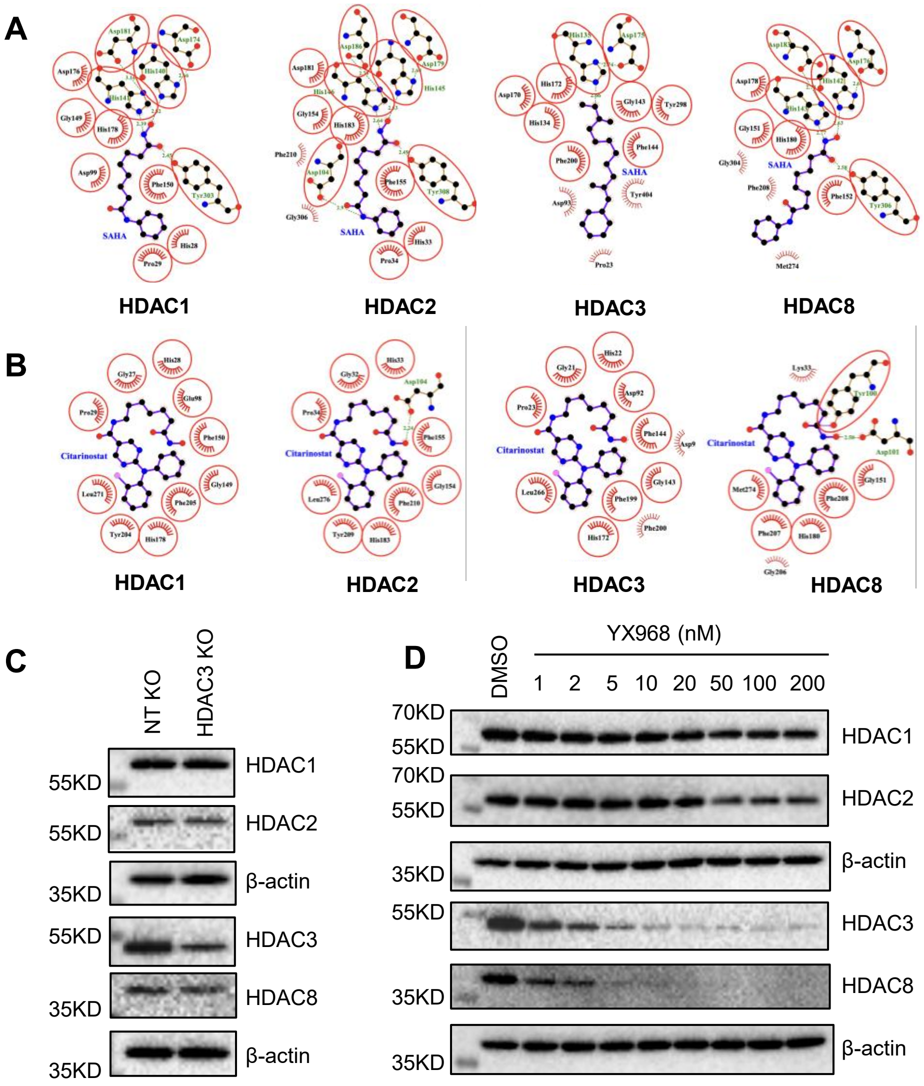


**Fig. S5. Citarinostat binds a conserved external site on Class I HDACs.**

LigPlot+ interaction diagrams for SAHA (**A**) and citarinostat (**B**) bound to HDAC1, HDAC2, HDAC3, and HDAC8. Hydrogen bonds are shown as green dashed lines and hydrophobic contacts as red spokes. Interacting residues within 4 Å of the ligand are labeled for each isoform. Residues common to all four HDACs are circled, highlighting the conserved elements of the canonical hydroxamate binding mode. (**C**) HDAC3 was knocked out in 2D10 cells via CRISPR-Cas9, using a non-targeting gRNA as the control, and the cells were cultured for five days. (**D**) In a separate experiment, 2D10 cells were pretreated for 24 h with DMSO or 20 nM YX968, an HDAC3/8 dual PROTAC. Total protein was extracted from cells in both experimental series and analyzed by western blot to assess the expression of various HDAC isoforms.

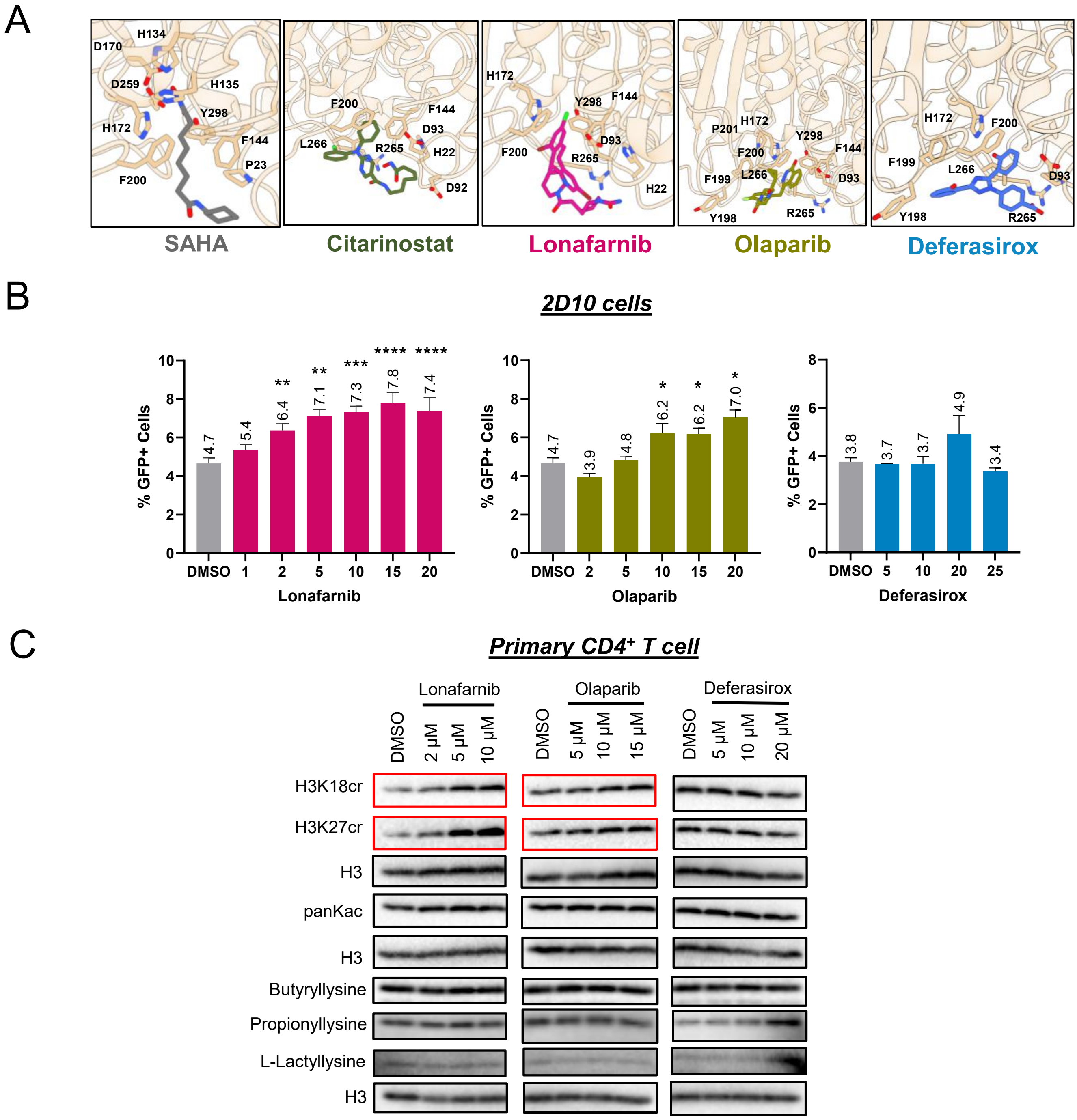


**Fig. S6. Virtual screening of selective HDCRi in FDA-approved drugs.**

(**A**) Lonafarnib, Olaparib, and Deferasirox were docked against the AI-generated enzyme pocket of HDAC3. (**B**) 2D10 cells were treated with Lonafarnib, Olaparib, and Deferasirox at the indicated concentrations for 24 hours. The level of GFP expression was measured by flow cytometry (n=6). (**C**) Primary CD4$^+$ T cells were treated with Lonafarnib, Olaparib, and Deferasirox for 1 hour. Total histone 3 and histone PTMs were detected by western blot.

A

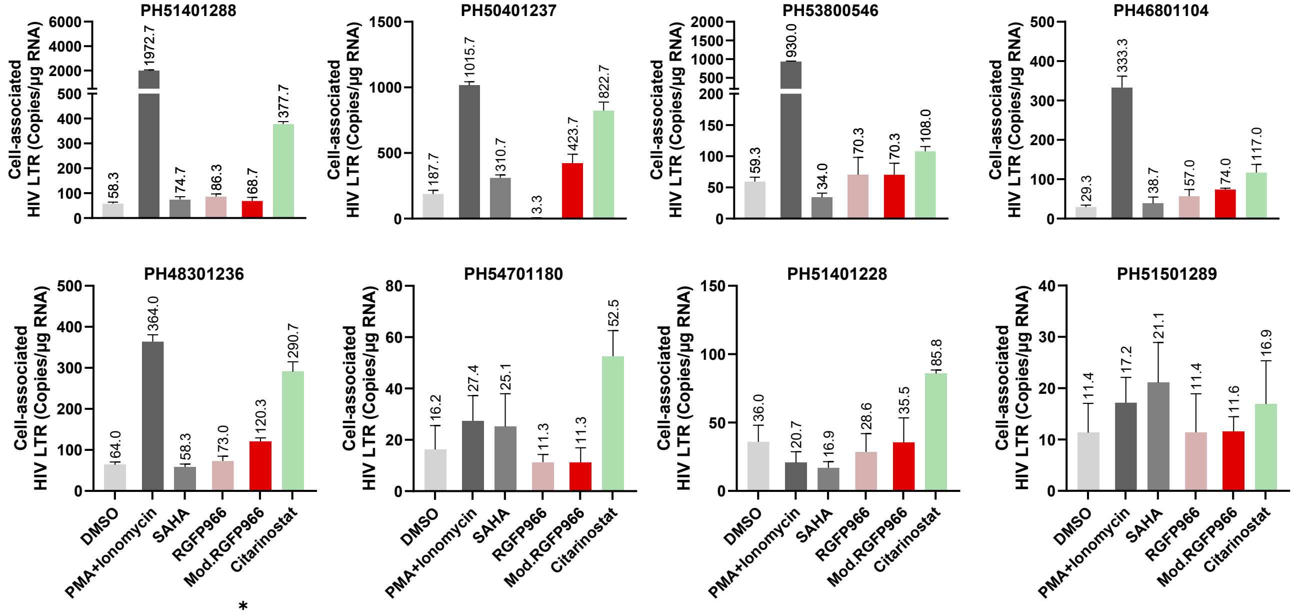


B

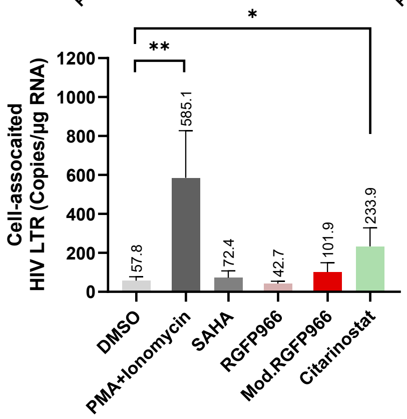


**Fig. S7. Selective HDCRi potently disrupts latent HIV in resting CD4+ T cells isolated from PWH on suppressive ART.**

Peripheral resting CD4+ T cells were isolated from 8 people with HIV on ART and treated with the indicated compounds for 24 hours. (**A**) The cell-associated HIV LTR levels were measured by ddPCR, with results shown individually for each donor, including triplicate ddPCR data. (**B**) A combined summary of the triplicate ddPCR results from all donors (n = 8) are presented as averages.

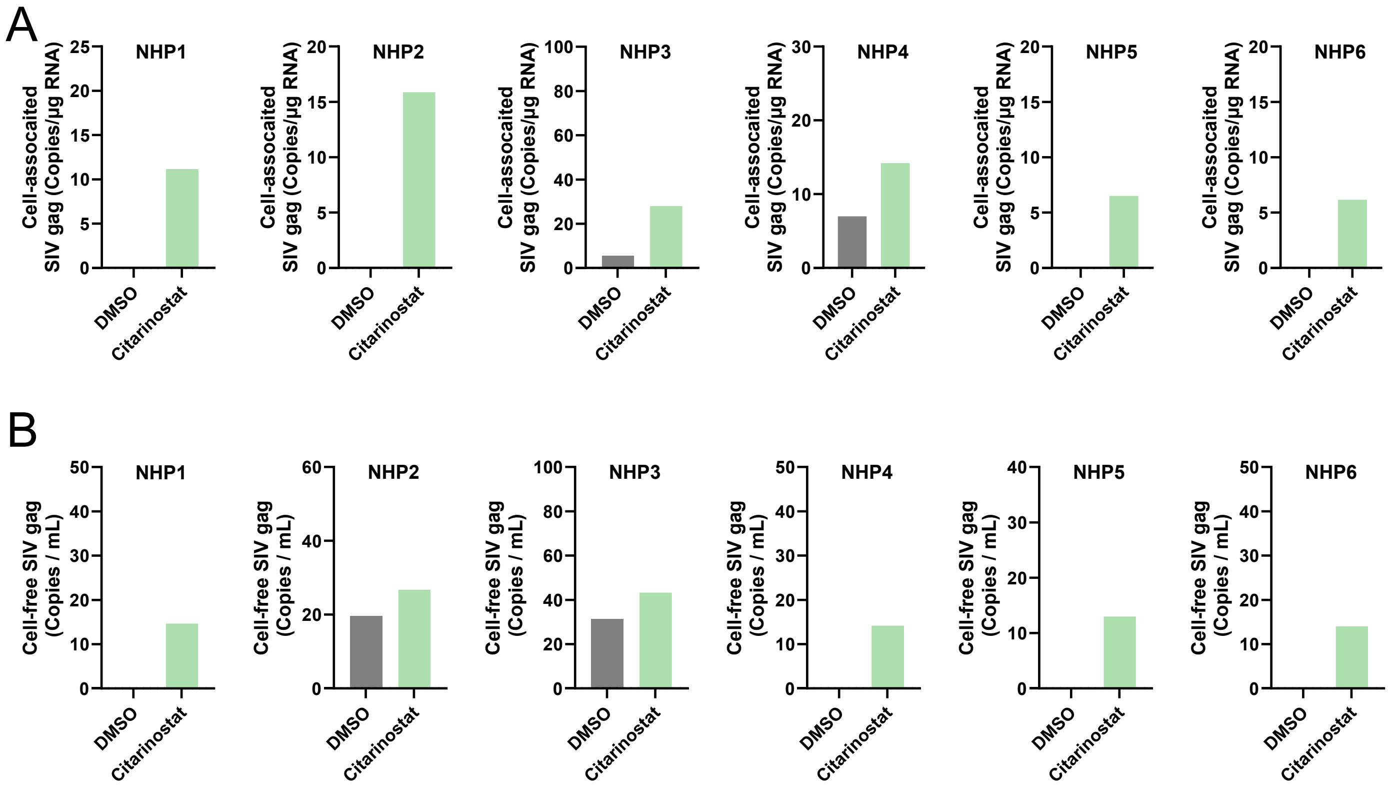


**Fig. S8. Selective HDCRi disrupts latent SIV isolated from NHPs.**

Brain MG from six NHPs were treated with citarinostat 5 µM or DMSO control. After 24 hours, the drugs were removed, and cells were cultured for another 6 days. Cells and culture supernatants were harvested for cell-associated (**A**) and cell-free (**B**) SIV RNA measurement by RT-ddPCR.

**Table S1. Characterization of the NHP**

| Sources | Animal ID | Gender | Age (yr) | pVL (copies/mL) | CD4 counts (cells/μL) |
|---|---|---|---|---|---|
| CNPRC | NHP 1 | F | 10 | 400 | 288 |
| CNPRC | NHP 2 | M | 6 | 40 | 275 |
| CNPRC | NHP 3 | F | 6 | 290 | 487 |
| ONPRC | NHP 4 | M | 18 | 600 | 403 |
| ONPRC | NHP 5 | F | 6 | 72 | 506 |
| ONPRC | NHP 6 | M | 8 | 25 | 342 |

CNPRC, California National Primate Research Center; ONPRC, Oregon National Primate Research Center; NHP, non-human private; M, male; F, female; pVL, plasma viral load.

**Table S2. Key Resources**

| REAGENT or RESOURCE | SOURCE | IDENTIFIER |
| --- | --- | --- |
| Antibodies | | |
| anti-NF-κB2 p100/p52 | Cell Signaling | Cat# 15003 |
| anti-ENL | Cell Signaling | Cat# 14893 |
| anti-CDYL | Gene Tex | Cat# GTX117708 |
| anti-β-Actin | Cell Signaling | Cat# 4970 |
| anti-α-Tubulin | Cell Signaling | Cat# 2144 |
| anti-Acetyl-α-Tubulin | Cell Signaling | Cat# 3971 |
| anti-HDAC1 | Cell Signaling | Cat# 34589 |
| anti-HDAC2 | Cell Signaling | Cat# 57156 |
| anti-HDAC3 | Cell Signaling | Cat# 85057 |
| anti-HDAC8 | Cell Signaling | Cat# 66042 |
| Histone H3 Antibody | Cell Signaling | Cat# 2650 |
| anti-H3K18Cr | PTM-Bio | Cat# PTM-7252 |
| anti-H3K27Cr | PTM-Bio | Cat# PTM-545RM |
| Anti-Histone H3 (acetyl K9 + K14 + K18 + K23 + K27) | Abcam | Cat# ab47915 |
| Normal rabbit IgG | Cell Signaling | Cat# 2729 |
| Anti-mouse IgG, HRP-linked Antibody | Cell Signaling | Cat# 7076 |
| Anti-rabbit IgG, HRP-linked Antibody | Cell Signaling | Cat# 7074 |
| Biological samples | | |
| Leukapheresis from HIV-infected individuals | University of North Carolina Hospital | N/A |
| HIV-negative primary CD4+ T cells | University of California, Los Angeles | N/A |
| Chemicals and recombinant proteins | | |
| SAHA | Selleck chemicals | Cat# S1047 |
| Suberoyl bis-hydroxamic acid (SBHA) | MedChemExpress | Cat# HY-W009776 |
| M344 | Selleck chemicals | Cat# S2779 |
| MS275 | Selleck chemicals | Cat# S1053 |
| Apicidin | Sigma-Aldrich | Cat# A8851 |
| crotonic acid | Sigma-Aldrich | Cat# 107-93-7 |
| splitomicin | Selleck chemicals | Cat# S7593 |
| RG2833 | Selleck chemicals | Cat# S7292 |
| RGFP966 | Selleck chemicals | Cat# S7229 |
| BG45 | Selleck chemicals | Cat# S7689 |
| Mocetinostat | Selleck chemicals | Cat# S1122 |
| Modified RGFP966 | Dr. Christopher Aquino | N/A |
| Modified Mocetinostat | Dr. Christopher Aquino | N/A |
| Citarinostat | Selleck chemicals | Cat# S8464 |
| Tucidinostat | Selleck chemicals | Cat# S8567 |
| CUDC-101 | Selleck chemicals | Cat# S1194 |
| Quisinostat | Selleck chemicals | Cat# S1096 |
| CUDC907 | Selleck chemicals | Cat# S2759 |
| YX968 | MedChemExpress | Cat# HY-164233 |
| Lonafarnib | Cayman Chemical | Cat# SML1457 |
| Olaparib | Cayman Chemical | Cat# SML3705 |
| Deferasirox | Cayman Chemical | Cat# SML2673 |
| Recombinant Human HDAC2 protein | Abcam | Cat# ab101662 |
| HDAC3/NCOR1 complex (human), (recombinant) | Enzo | Cat# BML-SE515 |
| Recombinant HDAC8 protein, His-Tag | Active motif | Cat# 31566 |
| Crotonoyl coenzyme A trilithium salt | Sigma-Aldrich | Cat# 28007 |
| Acetyl-Coenzyme A | Roche | Cat# ACOA-RO |
| Recombinant Histone H3.1 (Human) | Active motif | Cat# 31294 |
| p300 (catalytic domain) (human) | Enzo | Cat# BML-SE451 |
| NuPAGE™ LDS Sample Buffer (4X) | Invitrogen | Cat# NP0007 |

| | | |
|---|---|---|
| Seradigm, Premium Grade FBS | Avantor | Cat# 97068-085 |
| Penicillin-Streptomycin | Gibco | Cat# 15140122 |
| L-Glutamine | Gibco | Cat# 25030081 |
| HEPES | Gibco | Cat# 15630106 |
| Sodium Pyruvate | Gibco | Cat# 11360070 |
| Recombinant Human IL-2 | PeproTech | Cat# 200-02 |
| anti-CD3/CD28 dynabeads | Gibco | Cat# 11132D |
| DNase I | Invitrogen | Cat# 18047019 |
| SuperScript IV First-Strand Synthesis System | Invitrogen | Cat# 18091050 |
| Random primers | Invitrogen | Cat# 48190011 |
| LIVE/DEAD™ Fixable Far Red Dead Cell Stain Kit | Invitrogen | Cat# L34973 |
| RIPA lysis buffer | Sigma-Aldrich | Cat# R0278 |
| Protease/Phosphatase Inhibitor Cocktail (100X) | Cell signaling | Cat# 5872 |
| EpiQuik Total Histone Extraction Kit | EpigenTek | Cat# OP-0006-100 |
| Pierce™ 16% Formaldehyde (w/v), Methanol-free | Thermo Scientific™ | Cat# 28906 |
| PBS | Gibco | Cat# 14190-250 |
| NaCl (5 M), RNase-free | Invitrogen | Cat# AM9759 |
| IGEPAL® CA-630 (NP40) | Sigma-Aldrich | Cat# I8896 |
| Triton™ X-100 | Sigma-Aldrich | Cat# T8787 |
| Tris (1 M), pH 8.0, RNase-free | Invitrogen | Cat# AM9856 |
| EDTA (0.5 M), pH 8.0, RNase-free | Invitrogen | Cat# AM9260G |
| EGTA 0.5M, pH 8.0, Sterile | BioWORLD | Cat# 40520008 |
| SDS, 20% Solution | ThermoFisher | Cat# AM9820 |
| Nanodroplet cavitation reagent | MegaShear, Triangle Biotechnology | Cat# CS101-1000 |
| **Critical commercial assays** | | |
| Custom EasySep™ Human Resting CD4+ T Cell Isolation Kit | Stemcell Tech. | N/A |
| RNeasy mini kit | Qiagen | Cat# 74106 |
| TaqMan™ Universal PCR Master Mix | Applied Biosystems Inc. | Cat# 4304437 |
| Lipofectamine™ 3000 Transfection Reagent | Invitrogen | Cat# L3000015 |
| Luciferase Assay System | Promega | Cat# E1501 |
| ChIP Clean & Concentration kit | Zymo Research | Cat# D5205 |
| SYBR™ Green Universal Master Mix | Applied Biosystems Inc. | Cat# 4309155 |
| EasySep Release Human CD3 Positive Selection Kit | STEMCELL Technologies | Cat# 17751 |
| CD3 MicroBead Kit, nonhuman primate | Miltenyi Biotec | Cat# 130-092-012 |
| CD11b MicroBeads, human and mouse | Miltenyi Biotec | Cat# 130-049-601 |
| CD11b MicroBeads, NHP | Miltenyi Biotec | Cat# 130-091-100 |
| QIAamp viral RNA mini kit | Zymo Research | Cat# 52904 |
| SE Cell Line 4D-Nucleofector® X Kit S | LONZA | Cat# V4XC-1032 |
| **Experimental models: Cell lines** | | |
| 2D10 | Jonathan Karn lab | N/A |
| TZM-bl luciferase reporter cells | NIH HIV Reagent Repository | N/A |
| **Experimental models: Organisms/strains** | | |
| Primary CD4+ T cell infection model | Guochun Jiang lab | SF162 |
| Primary CD4+ T cell model of latency | Ed Browne lab | N/A |
| **Oligonucleotides** | | |
| HIV LTR probe: FAMCCA GAG TCA CAC AAC AGA CGG GCA CAT AMRA | IDT | Steven A. Yukl, et al. 2018 (81) |
| HIV LTR sense: GCC TCA ATA AAG CTT GCC TTG A | IDT | Steven A. Yukl, et al. 2018 (81) |
| HIV LTR antisense: GGG CGC CAC TGC TAG AGA | IDT | Steven A. Yukl, et al. 2018 (81) |
| HIV gag probe: FAM/CT CTC TCC T/ZEN/T CTA GCC TCC GCT AGT /3IABkFQ/ | IDT | Malnati et al., 2008 (80) |
| HIV gag FWD: TACTGACGCTCTCGCACC | IDT | Malnati et al., 2008 (80) |
| HIV gag REV: TCTCGACGCAGGACTCG | IDT | Malnati et al., 2008 (80) |
| SIV gag FWD: GCAGGAACAACTAGTTCAGTAGATGA | IDT | Jiang Lab |

| | | |
|---|---|---|
| SIV gag REV: CCAGTTGGATCCATCTCCTGTAAAT | IDT | Jiang Lab |
| SDHA (Hs00188166_m1) | Thermo Scientific | Cat# 4331182 |
| Alt-R Cas9 Negative Control crRNA #1 | Integrated DNA Technologies | Alt-R CRISPR-Cas9 crRNA |
| crRNA Hs. Cas9.HDAC3.1. AA: UAUUUCUACGACCCCGACGU | Integrated DNA Technologies | Alt-R CRISPR-Cas9 crRNA |
| crRNA Hs. Cas9.HDAC3.1. AB: UCUUAUAGAGACCGUUAAGC | Integrated DNA Technologies | Alt-R CRISPR-Cas9 crRNA |
| crRNA Hs.Cas9.HDAC3.1.AC: AUGUCAAUGUAGAGCACCCG | Integrated DNA Technologies | Alt-R CRISPR-Cas9 crRNA |
| Software | | |
| FlowJo V10 | BD | https://www.flowjo.com/ |
| GraphPad Prism 10 | GraphPad Software Inc. | https://www.graphpad.com/ |
| QuantaSoft™ Analysis Pro | Bio-Rad | https://www.bio-rad.com/ |
| Plasmid | | |
| pcDNA3.1-Flag | Addgene | Plasmid# 208051 |
| HDAC3 Flag | Addgene | Plasmid# 13819 |
| HDAC3 (VRPP, Y298H, D57A, Q113A, R265A, R301A) | GenScript | N/A |
| pNL43Δ6-dreGFP plasmid | Robert Siliciano, Johns Hopkins University | N/A |
| psPAX2 | Addgene | Plasmid# 12260 |
| pMD2.G (VSV-G) | Addgene | Plasmid# 12259 |
